# Characterization of two 1,3-β-glucan-modifying enzymes from *Penicillium sumatraense* reveals new insights into 1,3-β-glucan metabolism of fungal saprotrophs

**DOI:** 10.1186/s13068-022-02233-8

**Published:** 2022-12-12

**Authors:** Valentina Scafati, Francesca Troilo, Sara Ponziani, Moira Giovannoni, Anna Scortica, Daniela Pontiggia, Francesco Angelucci, Adele Di Matteo, Benedetta Mattei, Manuel Benedetti

**Affiliations:** 1 Department of Life, Health and Environmental Sciences, University of L’Aquila, 67100 L’Aquila, Italy; 2 Institute of Molecular Biology and Pathology, CNR, P.Le Aldo Moro 5, 00185 Rome, Italy; 3 Department of Biology and Biotechnology “Charles Darwin”, Sapienza University of Rome, 00185 Rome, Italy

**Keywords:** *Penicillium*, Cell wall-modifying enzymes, 1,3-β-Glucan metabolism, Exo-1,3-β-glucanase, 1,3-β-Transglucanase, TIM-barrel

## Abstract

**Background:**

1,3-β-glucan is a polysaccharide widely distributed in the cell wall of several phylogenetically distant organisms, such as bacteria, fungi, plants and microalgae. The presence of highly active 1,3-β-glucanases in fungi evokes the biological question on how these organisms can efficiently metabolize exogenous sources of 1,3-β-glucan without incurring in autolysis.

**Results:**

To elucidate the molecular mechanisms at the basis of 1,3-β-glucan metabolism in fungal saprotrophs, the putative exo-1,3-β-glucanase G9376 and a truncated form of the putative glucan endo-1,3-β-glucosidase (ΔG7048) from *Penicillium sumatraense* AQ67100 were heterologously expressed in *Pichia pastoris* and characterized both in terms of activity and structure. G9376 efficiently converted laminarin and 1,3-β-glucan oligomers into glucose by acting as an exo-glycosidase, whereas G7048 displayed a 1,3-β-transglucanase/branching activity toward 1,3-β-glucan oligomers with a degree of polymerization higher than 5, making these oligomers more recalcitrant to the hydrolysis acted by exo-1,3-β-glucanase G9376. The X-ray crystallographic structure of the catalytic domain of G7048, solved at 1.9 Å of resolution, consists of a (β/α)_8_ TIM-barrel fold characteristic of all the GH17 family members. The catalytic site is in a V-shaped cleft containing the two conserved catalytic glutamic residues. Molecular features compatible with the activity of G7048 as 1,3-β-transglucanase are discussed.

**Conclusions:**

The antagonizing activity between ΔG7048 and G9376 indicates how opportunistic fungi belonging to *Penicillium* genus can feed on substrates similar for composition and structure to their own cell wall without incurring in a self-deleterious autohydrolysis.

**Supplementary Information:**

The online version contains supplementary material available at 10.1186/s13068-022-02233-8.

## Background

In recent years, fungal biotechnology has become an eco-friendly technology exploitable in industrial processing using fungal enzymes in substitution of polluting chemicals [1]. In this regard, fungal saprotrophs are a valuable source of Cell Wall Degrading Enzymes (CWDEs), highly relevant for the bioconversion of lignocellulosic waste materials into biofuel-related compounds [2–4]. Well-known CWDE-producers are fungi belonging to *Trichoderma* [5], *Aspergillus* [6] and *Penicillium* genera [7, 8]. These organisms degrade plant biomass by secreting arsenals of CWDEs whose synthesis can be stimulated by culturing fungi on agricultural wastes, thus lowering the production costs associated with fungal fermentation processes [4]. The opportunistic and saprotrophic nature of fungi is also evidenced in *Penicillium sumatraense* AQ67100, a filamentous fungus that feeds on plants [9], macro- [10] and micro-algae [3]; noteworthy, the cell wall of land plants and algae is very different both in terms of composition and structure [4], pointing to *P. sumatraense* as a highly versatile saprotroph. To assimilate *C. vulgaris* cells, *P. sumatraense* AQ67100 secretes an enzymatic arsenal mainly composed of proteases, 1,3-β-glucanases and glycosidases. Our attention was drawn to the 1,3-β-glucan degrading machinery of *P. sumatraense,* since 1,3-β-glucan, differently from plant-specific cell wall polysaccharides, such as cellulose, hemicellulose and pectin, is widely distributed in several phylogenetically distant organisms. Indeed, the cell wall of many microalgae [11–13], bacteria [14], yeasts, and fungi, including *Penicillium* species [15], contains 1,3-β-glucans to a different extent. This polysaccharide is also present as a storage sugar in brown macroalgae from *Laminaria* genus (i.e., a 1,3/1,6-β-mixed glucan referred to as laminarin, [16]) and as defense-induced cell wall polysaccharide in land-plants (i.e., a 1,3/1,6-β-mixed glucan referred to as callose, [17]). Consequently, 1,3-β-glucan acting enzymes are widely distributed in organisms from different kingdoms. For example, 1,3-β-glucanases are produced by plants to counterattack fungal pathogens during infection [18] or are produced by molluscs and insects to assimilate exogenous 1,3-β-glucan [19–22]. However, the presence of highly active 1,3-β-glucanases in fungi evokes the biological question on how an organism with a cell wall composed of 1,3-β-glucan can efficiently metabolize an exogenous source of 1,3-β-glucan without incurring in a self-deleterious autohydrolysis. Probably, 1,3-β-glucan acting enzymes evolved in two distinct categories: (i) 1,3-β-glucan degrading enzymes, to degrade exogenous 1,3-β-glucan, and (ii) 1,3-β-glucan modifying enzymes, to make endogenous 1,3-β-glucan more resistant against the degrading action of the first category of enzymes.

To provide new insight into the glucan metabolism in the *Penicillium* genus, we focused on two enzymes (named G9376 and G7048) that have been previously identified in the culture filtrate of *P. sumatraense* AQ67100 fed with *C. vulgaris* biomass [3]. Based on prediction software analysis, the selected enzymes G9376 and G7048 correspond to a putative exo-1,3-β-glucanase and a putative glucan endo-1,3-β-glucosidase eglC, respectively. In accordance with the classification from the CAZy database (http://www.cazy.org/), G9376 belongs to the glycoside hydrolase 16 (GH16) family (http://www.cazy.org/GH16.html), whereas G7048 belongs to the GH17 family (http://www.cazy.org/GH17.html). Based on their distinctive features, i.e., different abundance in the presence of *C. vulgaris* biomass and classification in different GH families, these enzymes were selected for recombinant expression in *Pichia pastoris* to perform enzymatic and structural characterization and investigate the mechanisms underlying 1,3-β-glucan metabolism in fungal saprotrophs.

Our results showed that G9376 is indeed an exo-1,3-β-glucanase active on 1,3-β-glucan oligomers and laminarin, characterized by a high thermostability. On the other hand, in contrast to its predicted function, G7048 acts as a 1,3-β-transglucanase active on 1,3-β-glucan oligomers with a degree of polymerization (DP) higher than 5. The X-ray structure of the catalytic domain of G7048 (ΔG7048) showed that it consists of the (*β*/*α*)_8_ TIM-barrel fold characteristic of all GH17 family members and a V-shaped catalytic cleft containing the two conserved catalytic glutamic residues. Notably, ΔG7048 structure reveals features compatible with 1,3-β-glucanosyltransferase activity toward large sugar substrates.

## Results

### Heterologous expression of G9376 and ΔG7048 in *P. pastoris*

*P. pastoris* was selected as expression host for the recombinant production of the two putative 1,3-β-glucan modifying enzymes from the fungus *P. sumatraense* AQ67100 (i.e., G9376 and G7048) (Additional File 1: Data S1; [3]). Unfortunately, the recombinant expression of the entire G7048 (aa. 1–497) failed, probably due to the presence of a C-terminal unstructured Gly- and Ser-rich region with no relevant catalytic function (aa. 309–497). Moreover, since this region has been predicted to bind a glycosylphosphatidylinositol (GPI) tail for the anchoring of G7048 to the cell membrane (Additional File 1: Data S1b), we decided to express a truncated variant of the protein encompassing the first 308 residues (hereafter referred to as ΔG7048). G9376 and ΔG7048 were expressed as secreted proteins and fused to a myc-epitope and a poly-histidine-tag at their C-terminus (Additional File 1: Data S2). It is worth mentioning that yeasts can N-glycosylate recombinant proteins at different extent, potentially affecting protein properties [4]; in our instance, five potential N-glycosylation sites were predicted for G9376 and one for ΔG7048 (the latter was also detected in the crystal structure, *see* Methods 8) (Additional File 1: Data S2). The recombinant enzymes G9376 and ΔG7048 were successfully purified by IMAC chromatography (purity > 95% (Additional File 2: Figure S1) with yields of 1.8 and 0.85 mg/L for G9376 and ΔG7048, respectively. The structural integrity of the purified proteins was evaluated by Far-UV circular dichroism (CD) spectroscopy revealing that both proteins were expressed in a folded state (Additional File 2: Figure S2). Interestingly, thermal denaturation monitored by CD spectroscopy highlighted a different thermal stability for the two enzymes: while ΔG7048 showed a cooperative sigmoidal denaturation profile with an apparent Tm of 55 °C, G9376 was more stable and retained its native conformation up to 75 °C (Additional File 2: Figure S2).

### g9376 encodes an exo-1,3-β-glucanase active on 1,3-β-glucan oligomers and laminarin.

The gene g9376 was predicted to encode a putative exo-1,3-β-glucanase [3]. To effectively characterize G9376 in vitro in terms of catalytic activity and substrate specificity, we first tested its ability to hydrolyze different di- and polysaccharides including 1,3-β-linked saccharides. As reported in (Table 1), the enzyme showed the highest activity toward the laminarin from *L. digitata,* a 1,3-/1,6-β-mixed glucan of brown algae, and the 1,3-β-D-laminaripentaitol borohydride (LAM5ol), a 1,3-β-glucan pentamer with a reduced C1-end (specific activity of 72.1 and 18.9 U/mg, respectively), while it did not act as laminaribiohydrolase having no activity toward *p*-nitrophenyl–β-laminaribioside (*p*NPLAM2) (Table 1).

**Table 1:** Specific activity of exo-1,3-β-glucanase G9376 toward different substrates

Substrate	Specific activity (U/mg)
*p*NPGlc	–
*p*NPGal	–
*p*NPLAM2	–
PGA	–
CMC	0.053 ± 0.005
Arabinoxylan	–
LAM5ol	18.9 ± 0.05
Laminarin	72.1 ± 0.23

Enzyme activity, expressed as Units (µmol/min) per mg of enzyme, was evaluated at 25 °C and pH 5. Units are expressed as µmol reducing ends (for PGA, CMC, Arabinoxylan, LAM5ol and laminarin substrates) and µmol *p-*nitrophenol (for *p*NPGLc, *p*NPGal and *p*NPLAM2 substrates) released per minute. Values are mean ± SD, *n* = 3; — no activity detected

*pNPGlc* *p*-nitrophenyl-β-D-glucopyranoside, *pNPGal* *p*-nitrophenyl-β-D-galactopyranoside; *pNPLAM2* *p*-nitrophenyl-β-D-laminaribioside, *CMC* carboxymethyl-cellulose, *PGA* polygalacturonic acid, *LAM5ol* 1,3-β-D-laminaripentaitol borohydride

**Fig. 1:**
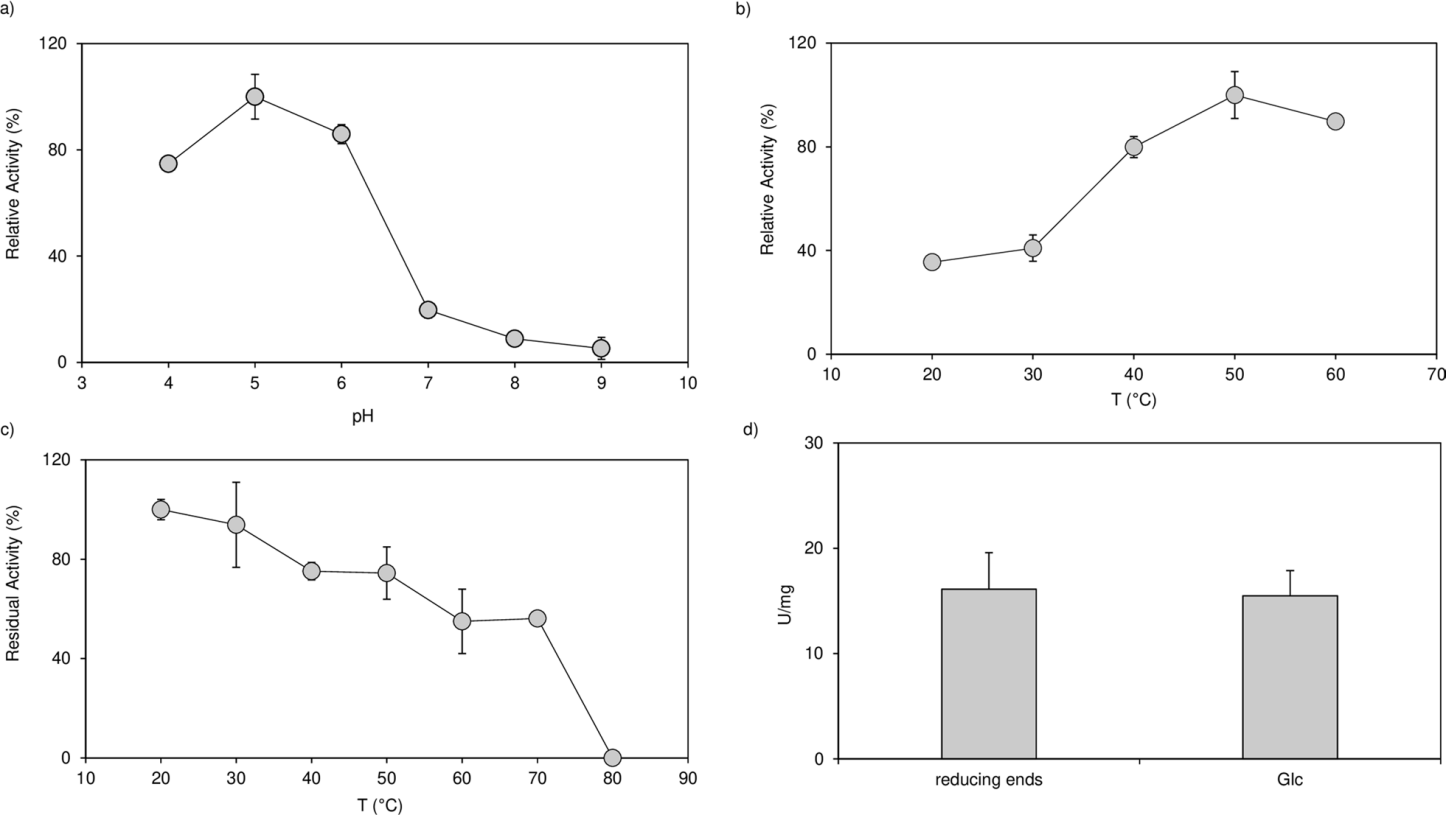
Enzymatic characterization of exo-1,3-β-glucanase G9376. (**a**) pH- and (**b**) temperature-dependent activity of exo-1,3-β-glucanase G9376 toward 0.2% (*w/v*) laminarin as determined by the reducing sugar assay. (**c**) Residual activity of exo-1,3-β-glucanase G9376 toward 0.2% (*w/v*) laminarin after 1 h-incubation at different temperatures as determined by the reducing sugar assay. (**d**) Specific activity expressed as Units (µmol reducing end/min, µmol Glc/min) per mg of exo-1,3-β-glucanase G9376 toward LAM5ol. The amounts of reducing ends and Glc were determined by the reducing sugar and GO-POD assays, respectively. Values are mean ± SD, *n *= 3. Analyses shown in (**b**–**d**) were performed at pH 5.0. [LAM5ol, 1,3-β-D-laminaripentaitol borohydride; Glc, D-Glucose]

Subsequently, the pH- and temperature-dependent activity of G9376 was investigated using laminarin as model substrate. As reported in Fig. 1 the enzyme can hydrolyze the substrate in a pH range between 4 and 6 (with an optimum around pH 5) and with a temperature optimum around 50 °C (Fig. 1a–b). In accordance with the marked thermostability observed by CD analysis (Additional File 2: Figure S2), the activity of G9376 was only reduced by 50% upon 1 h incubation at 70 °C (Fig. 1c).

**Fig. 2:**
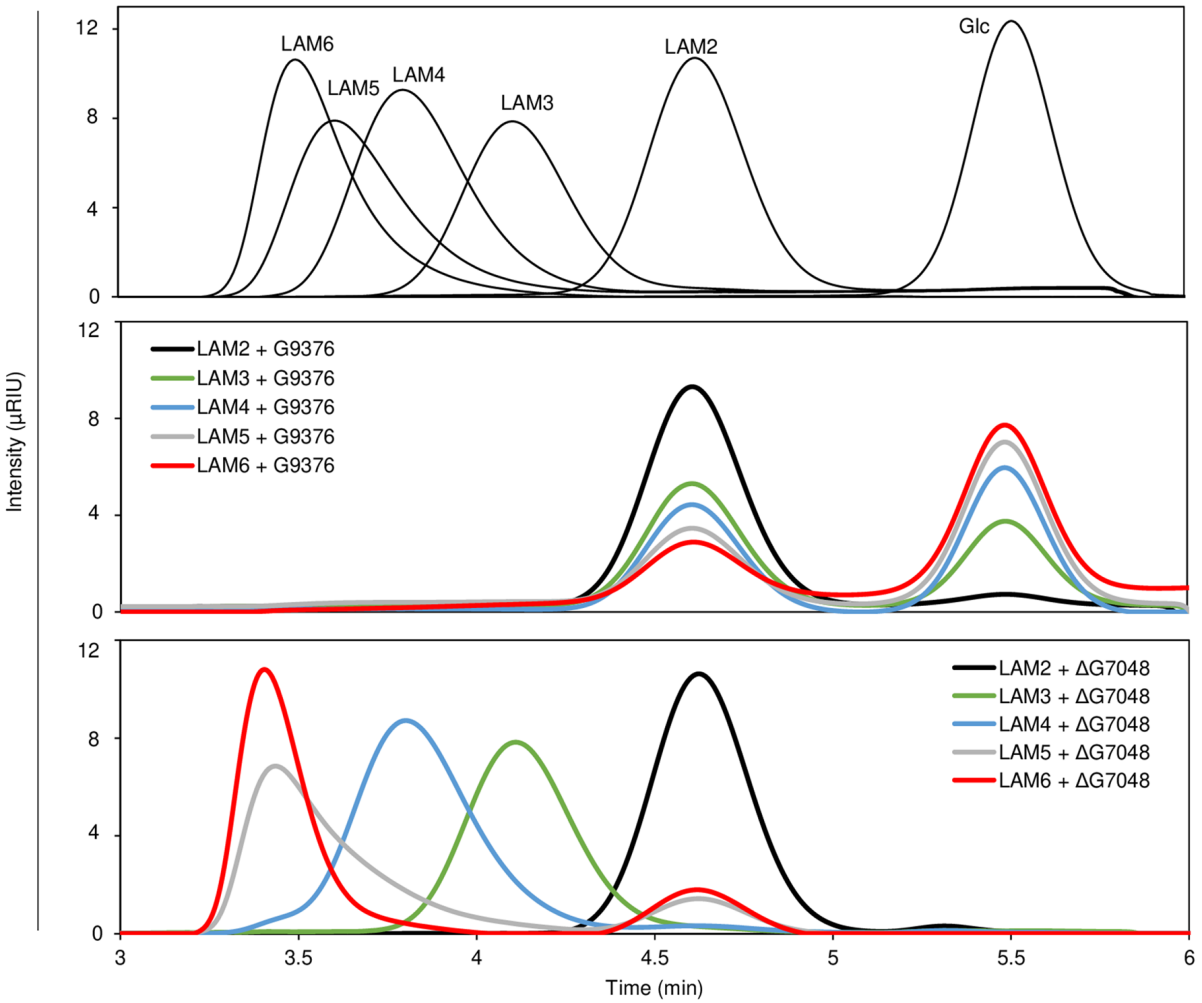
Analysis of degradation products obtained from different 1,3-β-glucan oligomers upon incubation with exo-1,3-β-glucanase G9376 and the enzyme ΔG7048**.** Chromatographic analysis of equal amounts of five different 1,3-β-glucan oligomers (*upper panel*) alone, (*middle panel*) upon 1 h incubation with exo-1,3-β-glucanase G9376 and (*lower panel*) upon 24 h incubation with the enzyme ΔG7048. In the upper panel, glucose was also analyzed. [*Glc D-Glucose*, *LAM2* laminaribiose, *LAM3* laminaritriose, *LAM4*, laminaritetraose, *LAM5* laminaripentaose, *LAM6* laminarihexaose]

Moreover, to ascertain whether G9376 catalyzes 1,3-β-glucan hydrolysis with an exo or endo mode of action, we compared the amount of D-Glucose (Glc) and reducing ends generated over time by G9376 using LAM5ol as substrate. The catalytic products resulted in a comparable amount of reducing ends and Glc (Fig. 1d), thus demonstrating that G9376 indeed acts as a glucanase with an exo-mode of action. HPLC chromatographic analysis showed that the enzyme hydrolyzes 1,3-β-glucan oligosaccharides that have a minimum of three units by releasing two end-products, i.e., Glc and laminaribiose (LAM2) (Fig. 2—*Middle panel)*. Moreover, the chromatographic analysis of the degradation products obtained using LAM5ol as a substrate revealed that Glc was released from the non-reducing end of the oligomer, since it accumulated together with two LAM-oligomers with borohydride-reduced C1-ends, i.e., 1,3-β-D-laminaritetraitol borohydride (LAM4ol) and 1,3-β-D-laminaritriitol borohydride (LAM3ol) (Additional File 2: Figure S3). To better identify the type of degradation products released from LAM5ol, the enzymatic reaction was also analyzed at different incubation times by HPAEC–PAD (Additional File 2: Figure S4). This analysis revealed that G9376 released Glc from the non-reducing end of LAM5ol by forming LAM4ol and LAM3ol as intermediate and end product, respectively (Additional File 2: Figure S4). This result was in accordance with the higher ratios [Glc: LAM2] observed for the hydrolysis of longer LAM-oligomers (Fig. 2—*Middle panel*), pointing to LAM2 as the residual (reducing) end of each 1,3-β-glucan oligomer that accumulates downstream of the action of exo-1,3-β-glucanase G9376. Overall, our results clearly showed that G9376 is an exo-1,3-β-glucanase sequentially cleaving glucose residues from the non-reducing end of 1,3-β-glucan oligosaccharides comprising at least three glycosidic units [(Glc1–3βGlc1–3βGlc)-n].

### g7048 encodes a transglucanase active on 1,3-β-glucan oligomers with a DP higher than 5.

The gene g7048 was predicted to encode a glucan endo-1,3-β-glucosidase [3]; therefore, the catalytic activity of ΔG7048 was analyzed toward the same substrates used for the enzymatic characterization of G9376. Surprisingly, ΔG7048 did not show any relevant hydrolase activity toward each substrate tested (data not shown), suggesting that the two proteins have different enzymatic activities. To unveil the enzymatic properties of ΔG7048, HPLC analysis was carried out using different 1,3-β-glucan oligomers as substrates. When laminaripentaose (LAM5) and laminarihexaose (LAM6) were used, the action of ΔG7048 gave rise to a small peak with a retention time compatible with LAM2 and one consistent with a larger oligomer with reduced retention time compared to that of the starting substrates. Interestingly, no changes in the substrate retention times were observed upon incubation of ΔG7048 with LAM2-4 (Fig. 2—*Lower panel*). These results suggested that ΔG7048 is active only on LAM-oligomers with a DP ≥ 5 and that its action results in an increased length of the substrate by forming at least one hybrid oligosaccharide. In accordance with what reported in Frankova et al. [23], the concomitant presence of an oligomer larger than the substrate and of LAM2 as enzymatic products suggested that ΔG7048 acts as a 1,3-β-transglucanase. Following this hypothesis, ΔG7048 should act by converting LAMX (where X ≥ 5) into LAM(X-2) and LAM2, and then by attaching LAM(X-2) to a new LAMX-acceptor.

To confirm this mode of action, LAM5 was incubated with ΔG7048 for different times and the reaction products were analyzed by HPLC. HPLC analysis revealed that hybrid oligosaccharide(s) accumulated together with LAM2 (Additional File 2: Figure S5). However, since the larger product eluted with a retention time out of the optimal separation range (Additional File 2: Figure S5), we carried out mass spectrometry analysis on the product/s obtained at the longest incubation time (24 h) to ascertain its exact DP and the eventual presence of multimers. MALDI–TOF mass spectrometry showed a major peak corresponding to an oligosaccharide composed of 8 Glc units (m/z: 1337.5, hereafter referred to as hybrid octamer or “H8”) and small amounts of longer oligomers, named as “H11” and “H14”, characterized by mass increments of three Glc units compared to the hybrid octamer (Additional File 2: Figure S6—*Right panel*). This result indicated that ΔG7048 used the newly generated hybrid oligosaccharides as substrates for sequential LAM3 additions, thereby resulting in a + DP3-series formed by “LAM5”–“H8”–“H11”–“H14” (Additional File 2: Figure S6). Intriguingly, a less abundant series with mass increments of 3 Glc units was also observed starting with LAM6 (“LAM6”-“H9”-H12″) (Additional File 2: Figure S6—*Right panel*), an oligomer present as contaminant also in the commercial LAM5 (Additional File 2: Figure S6—*Left panel*). Overall, these findings indicate that ΔG7048 acts with a two-step mechanism in which the LAM5 substrate is first cleaved to LAM2 and LAM3, then LAM3 is bound to a new LAM5-acceptor via a transglycosylation step, generating a hybrid octamer (LAM5 + LAM3 = H8). Subsequently, ΔG7048 can use the hybrid octamer as an acceptor of a freshly cleaved LAM3 oligomer and the resulting hybrid undecamer (H8 + LAM3 = H11) as a new substrate in its turn. Indeed, our results clearly indicated that the active substrate of 1,3-β-transglucanase ΔG7048 must have a minimum DP of five units.

### Structural model of the hybrid octamer through the analysis of degradation products.

**Fig. 3:**
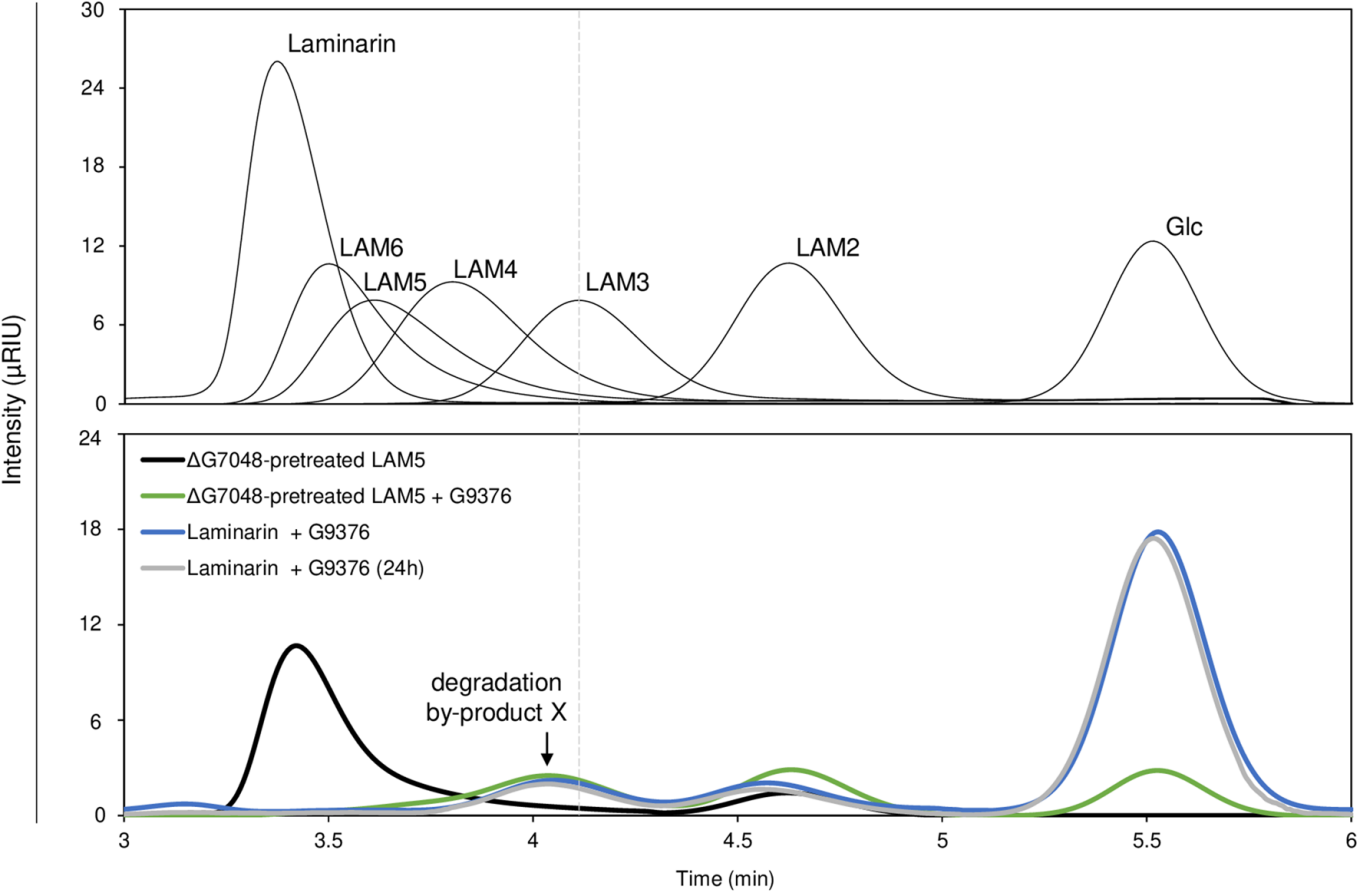
Analysis of degradation products obtained from a ΔG7048-pretreated LAM5 and laminarin upon incubation with exo-1,3-β-glucanase G9376. Chromatographic analysis of (*upper panel*) glucose, five different 1,3-β-glucan oligomers and laminarin, and of (*lower panel*) degradation products obtained from a ΔG7048-pretreated LAM5 upon 1 h-incubation with exo-1,3-β-glucanase G9376 (ΔG7048-pretreated LAM5 + G9376), and from laminarin upon 1 h- (Laminarin + G9376) and 24 h-incubation with exo-1,3-β-glucanase G9376 [Laminarin + G9376 (24 h)]. ΔG7048-pretreated LAM5 was analyzed as control. Black arrow indicates the peak corresponding to the degradation by-product X. [*Glc* D-Glucose, *LAM2* laminaribiose, *LAM3* laminaritriose, *LAM4* laminaritetraose, *LAM5* laminaripentaose, *LAM6* laminarihexaose]

To clarify the structure of the longer hybrid oligomers, we analyzed the products obtained from LAM5 pretreated with ΔG7048 (i.e., ΔG7048-pretreated LAM5) upon incubation with G9376 by HPLC. The analysis showed that, in addition to the conventional end-products obtained from 1,3-β-oligomers treated with exo-1,3-β-glucanase (Glc and LAM2) (Fig. 2*—Middle panel*), a novel peak appeared indicating an unknown degradation product (hereafter referred to as degradation “by-product X”). Notably, a peak with the same retention time was also detected when G9376 was incubated with laminarin, a storage polysaccharide from *L. digitata* composed of a 1,3/1,6-β-mixed glucan (Fig. 3). The enzymatic hydrolysis of degradation “by-product X” did not occur even after 24 h-incubation [*see* Laminarin + G9376 (24 h), Fig. 3] suggesting that the “by-product X” is a branched compound (therefore, uncleavable by G9376) and that ΔG7048 is responsible for such a branching activity. Next, to obtain additional information on the hybrid octamer, the same amounts of LAM5 and a ΔG7048-pretreated LAM5 were incubated with exo-1,3-β-glucanase G9376 and the resulting degradation products quantified by HPLC. In accordance with its exo-glycosidase activity, G9376 converted LAM5 into Glc and LAM2 with a molar ratio 3: 1 [Glc: LAM2] (Table 2); however, when incubated with a ΔG7048-pretreated LAM5, the amount of LAM2 remained almost the same, whereas the amount of Glc decreased to a molar ratio 1.5: 1 [Glc: LAM2] (Table 2). In accordance with these results, G9376 converted 2 molecules of LAM5 into 2 molecules of LAM2 and 6 molecules of Glc, and 2 molecules of ΔG7048-pretreated LAM5 into 2 molecules of LAM2, 3 molecules of Glc and 1 molecule of degradation by-product X that, necessarily, corresponded to a branched trimer, since it was formed at expense of 3 molecules of Glc (Table 2).

**Table 2:** Determination of degradation products obtained from a ΔG7048-pretreated LAM5 upon incubation with exo-1,3-β-glucanase G9376

	µg sugars			nmol sugars		
	ΔG7048-pretreated LAM5	LAM5 + G9376	ΔG7048-pretreated LAM5 + G9376	ΔG7048-pretreated LAM5	LAM5 + G9376	ΔG7048-pretreated LAM5 + G9376
Glc	–	6.2 ± 0.1	3.4 ± 0.1	–	34.3 ± 0.3	18.9 ± 0.5
LAM2	2.5 ± 0.1	4 ± 0.1	4.1 ± 0.1	7.4 ± 0.2	11.8 ± 0.1	12.1 ± 0.1
Degradation by-product X	–	–	2.7 ± 0.2	–	–	5.3 ± 0.4
Hybrid oligosaccharides	7.7 ± 0.1	–		5.9 ± 0.05	–	–
				Glc:LAM2	2.9	1.5

Amounts of degradation products (expressed as µg and nmol sugars) obtained from a ΔG7048-pretreated LAM5 (ΔG7048-pretreated LAM5 + G9376) and LAM5 (LAM5 + G9376) upon 1 h-incubation with exo-1,3-β-glucanase G9376 as determined by HPLC analysis. The amounts of products from a ΔG7048-pretreated LAM5 (ΔG7048-pretreated LAM5) are reported as control. Values are mean ± s.d. (*n* = 3). — not detected. [*Glc* D-Glucose *LAM2* laminaribiose]

**Fig. 4:**
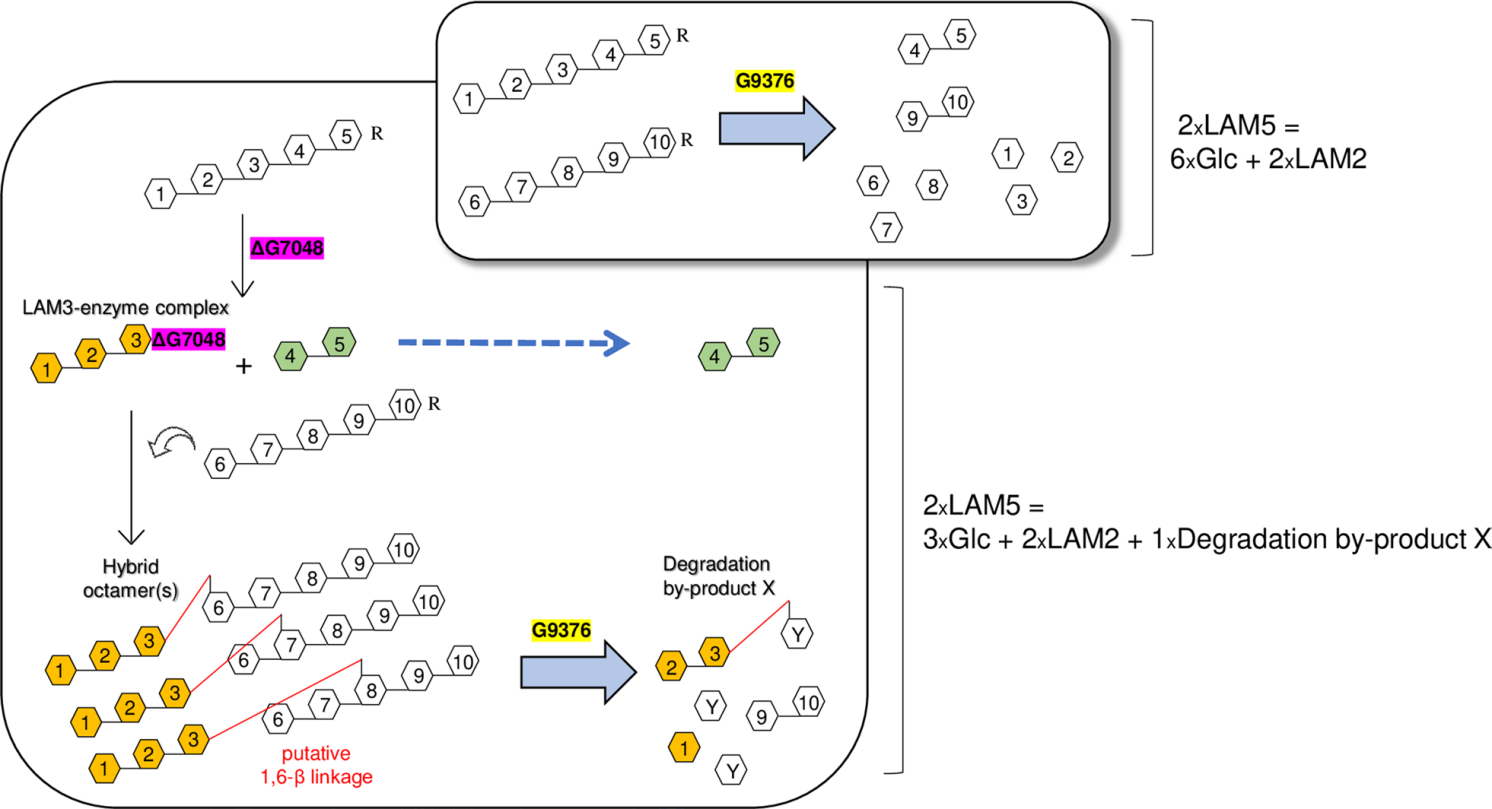
Scheme of the reaction catalyzed by 1,3-β-transglucanase ΔG7048 using LAM5 as a substrate, and degradation of the product(s) by exo-1,3-β-glucanase G9376. *Top panel*: hydrolysis catalyzed by exo-1,3-β-glucanase G9376 on LAM5 as deduced from the analyses shown in Figs. 1d, 2 and Additional File 2: Fig. S3. *Bottom panel*: transglycosylation catalyzed by 1,3-β-transglucanase ΔG7048 using LAM5 as substrate, and subsequent hydrolysis of the hybrid octamer(s) by exo-1,3-β-glucanase G9376. Red line indicates the putative 1,6-β-glycosidic linkage. The molar ratio of degradation products obtained from both enzymatic reactions (2xLAM5 + G9376) and (2xLAM5 + ΔG7048 + G9376) are reported on the right. The reducing end (R) of each LAM5 oligomer is also indicated [*Glc* D-Glucose, *LAM2* laminaribiose, *LAM3* laminaritriose, *LAM5* laminaripentaose; Y, residue n. 6 or n. 7 or n. 8]

A model of the formation of the hybrid octamer, based on the observed composition of the different reaction products, is reported in Fig. 4. According to our model, the hybrid octamer is composed of a linear 1,3-β-glucan pentamer attached to a trimer through a putative 1,6-β-linkage and can exist in three different isomers. Here, the type of glycosidic bond was suggested by the presence of degradation by-product X also in the G9376-treated laminarin (Fig. 3), a polysaccharide composed of 1,6/1,3-β-mixed glucan. The proposed models are the only structures in agreement with both the molar ratio of degradation products (Table 2) and the mode of action of G9376 (Fig. 2, Additional File 2: Figure S3). The analysis of degradation products clearly indicated that the branching activity of 1,3-β-transglucanase ΔG7048 on LAM5 reduced the hydrolyzing activity of exo-1,3-β-glucanase G9376 by 45% (Table 2). The latter conclusion was also corroborated by the high hydrolyzing activity displayed by exo-1,3-β-glucanase G9376 toward laminarin. Here, laminarin was efficiently converted into Glc, whereas the amount of residual branched by-product X was rather low (Fig. 3). Thus, the ratio [Glc: degradation by product X] was at least fivefold higher for laminarin than the hybrid octamer, clearly indicating that the branching activity of ΔG7048 strongly reduced the Glc-releasing activity of G9376 on 1,3-β-glucan oligomers (Fig. 3). Our results suggested also that exo-1,3-β-glucanase G9376 can still release Glc from the non-reducing end of a branched oligomer, provided that its main chain contains at least two consecutive 1,3-β-linkages (Table 2, Fig. 4).

### 3D-structure of ΔG7048 reveals important features for transglucanase activity.

**Fig. 5.**
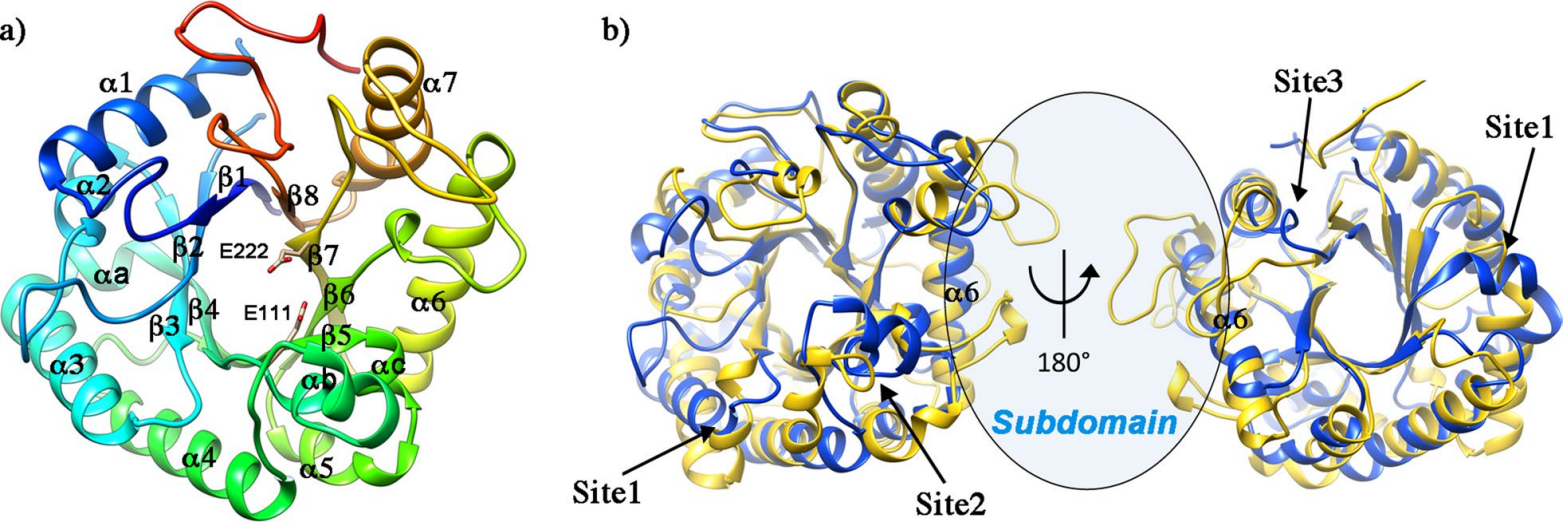
3D-structure of 1,3-β-transglucanase ΔG7048. **a** Rainbow colored cartoon of the structure of ΔG7048 with the catalytic residues in stick representation. **b** Structural superposition of ΔG7048 (blue) with the endo-1,3-β-glucanase of *S. tuberosum* (PDB: 3ur8, yellow)

To get insight into the molecular bases of the catalytic properties of ΔG7048, we undertook its structural characterization by X-ray crystallography (Table 3). Overall, the structure consists of a (*β*/*α*)_8_ TIM-barrel fold typical of the glycoside hydrolase GH17 family that includes both 1,3-β-glucanosyltransferase and 1,3-β-glucanases [24–26]. The structure comprises eight β-strands arranged in a parallel β-barrel surrounded by α-helices that are connected to the central barrel by loops of different lengths. The catalytic site is in a deep cleft containing the conserved glutamic catalytic residues (E111 and E222) at the center of the V-shaped substrate-binding region (Fig. 5a).

**Table 3:** X-ray data collection and refinement statistics for ΔG7048

Data collection	
Wavelength (Å)	1
Space group	P21212
Cell dimensions (Å)	a = 79.2 b = 87.3 c = 42.5
Resolution range (Å)	43.65–1.90 (1.94–1.90)^a^
CC(1/2) (%)	99.9 (95.8)^a^
I/sigma I	18.9 (4.6)^a^
Completeness (%)	100 (100)^a^
*Reflections*	
Total no. unique reflections	23,992 (1519)^a^
Multiplicity	11.4 (11.6)^a^
Refinement statistics	
R_work_/R_free_ (%)	0.16/0.20
Resolution range average B-factor (Å^2^) (no. of atoms)	43.65–1.90
Overall	30.62 (2502)
Protein	29.04 (2227)
Waters	37.31 (184)
Others	55.73 (91)
*RMSD*	
Bond length (Å)	0.006
Bond angle (°)	0.772
*Ramachandran* (%)	
Preferred/Allowed	98.94/1.06
PDB ID	8akp

^a^Values in parentheses are for the outer shell

**Fig. 6:**
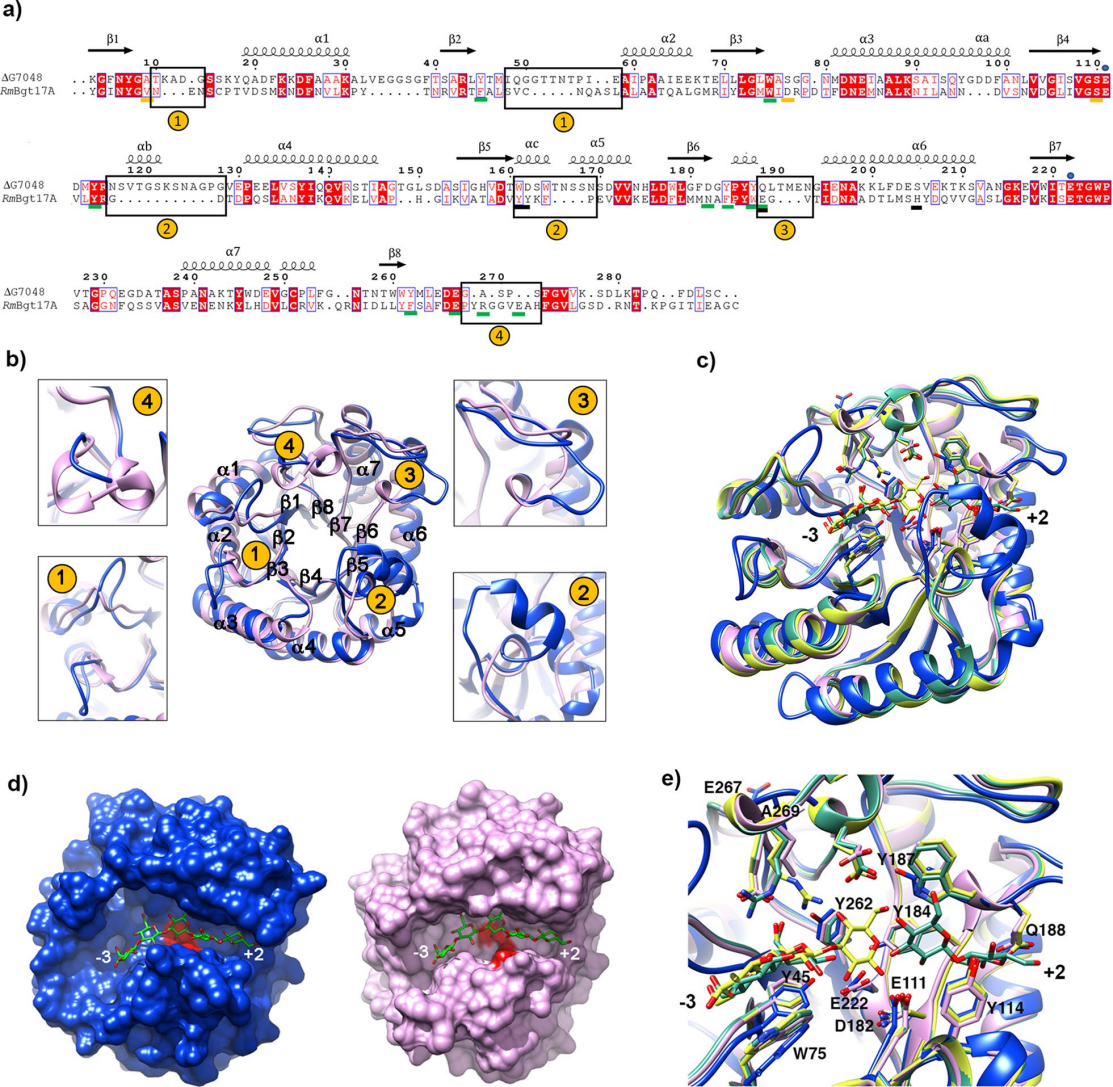
Comparison between ΔG7048 and *Rm*Bgt17A. **a** Structural-based sequence alignment between ΔG7048 and *Rm*Bgt17A. Identical residues are shown on a red background and conservatively mutated residues are shown in red on white background. The catalytic residues, E111 and E222, are marked with blue dots. Residues reported to be involved in direct or H_2_O-mediated interaction with the substrate in *Rm*Bgt17A are underlined in green and orange, respectively. Residues limiting the access to the catalytic cleft in *Rm*Bgt17A are underlined in black. Regions corresponding to the 4 sites described in panel b are indicated by black boxes. The sequences were aligned by Chimera and the figure was produced in *ESPript.* **b** Structural superposition of ΔG7048 (blue) with the ligand free form of *Rm*Bgt17A (PDB: 4wtp, pink). **c** structural superposition of ΔG7048 (blue) with (i) ligand free *Rm*Bgt17A (PDB: 4wtp, pink), (ii) LAM2*-Rm*Bgt17A (PDB: 4wtr, green) and (iii) LAM3-*Rm*Bgt17A (PDB: 4wts, yellow). **d** Surface representation of ΔG7048 (*left*) and *Rm*Bgt17A (PDB: 4wtp, *right*) with substrates from *Rm*Bgt17A structures (PDB: 4wtr, PDB: 4wts) located in the catalytic cleft without further docking experiment or energy minimization procedure. **e** Close view of the substrate binding region of structures superposed in **c**. Catalytic residues and those directly contacting the substrates in *Rm*Bgt17A with the corresponding ones in ΔG7048 are representing in sticks (numbering according to the ΔG7048 sequence)

Figure 5b reports the structural superposition of ΔG7048 with the potato endo-1,3-β-glucanase as an example of a classical GH17 member with glucanase activity (pdb: 3ur8, [27]). Superposition shows that the internal β-barrel is well conserved, while several differences are in the zone surrounding the active site. In particular, significant differences are in the region connecting β3 and β4 (site1) and in the area connecting β4 to α4 and β5 to α5 (site2)*.* Other differences comprise loops that connect the secondary structural elements, mainly those facing the active site apart from the loop connecting α7 and β8 on the back side of the protein (site3). An important feature of ΔG7048 is the absence of the so-called “subdomain” in the proximity of the helix α6 (Fig. 5b), which is present in other glycoside hydrolase of the GH17 family and has been proposed to provide subsites + 3, + 4 and + 5 for substrate binding in 1,3-β-glucanase [24]. Notably, ΔG7048 shares this peculiar feature with *Rm*Bgt17A, a protein belonging to the GH17 family, which acts as a β-1,3-glucanosyltransferase [26]. Indeed ΔG7048 and *Rm*Bgt17A structures superpose very well (rmsd = 1.1 Å between 188 pruned atoms and 2.9 Å across all pairs) (Fig. 6b). The secondary structural elements of the TIM-barrel are well conserved in the two structures; the most significant differences are localized in the areas surrounding the active site that shapes the substrate binding region; particularly: (i) in the loop connecting β1 to α1 and β2 to α2 (region 1), (ii) in the αb and αc helices (region 2), (iii) in the loop connecting the helix β6 and the α6 (region 3), and iv) in the C-terminal portion (region 4) (Fig. 6b). To analyze the putative substrate binding region in ΔG7048, we took advantage of the superposition of our structure with those of *Rm*Bgt17A in complex with LAM3 and LAM2 (PDB: 4wts; 4wtr [26]). As shown in Fig. 6c, d, the substrate binding pocket of ΔG7048 appears to be wider than that of *Rm*Bgt17A, mainly in the region upstream of the site3 of the sugar. Moreover, albeit ΔG7048 and *Rm*Bgt17A share a low amino acid identity (29%), both the catalytic glutamic residues (E111 and E222) and several aromatic residues pointed out by Qin and colleagues as involved in direct substrate binding in *Rm*Bgt17A [26] are conserved or conservatively mutated in ΔG7048 (Fig. 6a, e). Structural and mutagenesis studies have identified three of these residues (Y102, W157, and E158 in *Rm*Bgt17A) as crucial for transglycosylation activity toward hydrolytic activity [28]. In the ΔG7048 structure, their topological position is occupied by the residues Y114 (Y102), Y187 (W157), and Q188 (E158) (Fig. 6a, e and Additional File 2: Figure S7) which are likely to fulfill the same proposed role in the stabilization of the transition state at subsite − 1, + 1 and + 2 during the transglycosylation reaction step.

Furthermore, Qin and colleagues proposed that, in *Rm*Bgt17A, four bulky residues (Y135, Y136, E158, and H172) located around the helix α6 are responsible for blocking the catalytic cleft near the O_1_ of the + 2 glucosyl residue. In the ΔG7048 structure, their topological position is occupied by W161, D162, Q188 and S205 (Fig. 6a and Additional File 2: Figure S7) which, together with the structural elements in regions 2 and 3 (Fig. 6b) are likely to create a similar steric hindrance. Overall, structural characterization provides the molecular bases of the transglycosylation activity displayed by ΔG7048.

## Discussion

In addition to well-known species used in pharmaceutical industry as source of β-lactam antibiotics [29], *Penicillium* genus includes also opportunistic species that act as plant pathogens [30, 31] and as contaminants in the feed and food industry [32, 33]. In general, *Penicillium* species are efficient decomposers that contribute to the global carbon cycle thanks to their ability to feed on the most disparate organic sources due to their marked propension to saprotrophy [34]. This peculiar trophic versatility requires coordinated and balanced action between (i) an enzymatic arsenal capable of efficiently dismantling several organic substrates and (ii) an enzymatic system that preserves the fungus from the destructive potential of its own degrading enzymes. Here, we investigated two 1,3-β-glucan modifying enzymes (G9376 and G7048) previously identified in *P. sumatraense* AQ67100 culture [3]. Both proteins were produced as recombinant proteins in *P. pastoris* and subjected to enzymatic and structural characterization.

We showed that G9376 acts as an exo-1,3-β-glucanase, efficiently converting linear 1,3-β-glucan oligomers and more complex substrates, such as laminarin, into glucose, a carbon source easily assimilable. Noteworthy, to our knowledge, G9376 is the first exo-1,3-β-glucanase from *Penicillium* species so far characterized, since previous reports were limited to the analysis of expression level of 1,3-β-glucanases from *Penicillium* upon growth in different carbohydrate-supplemented media [35]. The marked thermostability of the exo-1,3-β-glucanase G9376 (Fig. 1c, Additional File 2: Figure S2b) makes this enzyme a good candidate as supplement for biomass-degrading blends of industrial relevance [36, 37].

For the characterization of G7048, due to difficulty in the expression of the entire protein, a truncated variant comprising the catalytic domain and lacking the C-terminal unstructured region (ΔG7048) was produced. Enzymatic analyses revealed that G7048 acts as a 1,3-β-transglucanase, rather than a glucanase as inferred by predictions, creating branched-glucans. A similar glucanosyltransferase branching activity was initially described in *S. cerevisiae* [38] and later in *Aspergillus fumigatus* [39]. Indeed, the catalytic domain of 1,3-β-transglucanase ΔG7048 has a high amino acid identity (68.4%) with that of 1,3-β-glucanosyltransferase 2 (Bgt2p) from *A. fumigatus* (Additional File 2: Figure S8). Notably, Bgt2p converts two LAMX oligomers (where X ≥ 5) into one LAM2 and one 1,6/1,3-β-branched glucan oligomer (DP = 2X-2), with the latter existing in two differently abundant isomers [40]. A similar transglucanase/branching activity is suggested also for G7048 (Fig. 4). In analogy with the mechanism proposed for Bgt2p [40], our results may support a retaining mechanism, where G7048 cleaves after the second glucosyl residue from the reducing end of LAM5 releasing LAM2, and then transfers the residual LAM3 to a second molecule of LAM5, here acting as an acceptor, generating a branched octamer (DP = 2 × 5− 2) (Fig. 4).

Structural characterization of ΔG7048 provided the molecular bases for its 1,3-β-transglucanase activity. Indeed, the protein not only adopts a (β/α)_8_ TIM-barrel fold, typical of GH17 family members, but it shares unique structural features with *Rm*Bgt17A, the only GH17 enzyme with a glucanosyltransferase activity whose structure has been solved [26]. The most important structural difference between ΔG7048/*Rm*Bgt17A and other GH17 members is the absence of a conserved “subdomain” region in the proximity of the helix α6 (Fig. 5b), that is responsible for the binding of substrates at site + 3, + 4 and + 5 [24]. Qin and colleagues [26] correlated the absence of this “subdomain” and the presence of 4 superficial bulky side chain residues around the + 2 subsite with the shape of the catalytic cleft of *Rm*Bgt17A that, in the first step of the transglycosylation reaction (i.e., cleavage of a 1,3-β-linkage), leads to the release of LAM2 as the product. A similar steric hindrance in ΔG7048 is guaranteed not only by some of these bulky residues (Additional File 2: Figure S7), but also by secondary structural elements (site 2 and 3 in Fig. 6b) which result in a cleft with a similar shape around the + 2 subsite, in accordance with the evidence that LAM2 is released by its enzymatic activity. The general catalytic mechanism of the GH17 family members consists of an acid/base reaction carried out by two glutamate residues acting as a proton donor and nucleophile, respectively. In G7048, the two catalytic residues (E111 and E222) are conserved, as well as some of the residues involved in the interaction with the substrate, e.g., W75, a residue already identified in *Rm*Bgt17A (W65 in *Rm*Bgt17A) and conserved in all glucanosyltransferases of the GH17 family, and Y114 (Y102 in *Rm*Bgt17A), a residue involved in stabilizing the transition state of the reaction toward transglycosylation [26]. Moreover, most of the other residues involved in substrate binding or proposed to be involved in the stabilization of the transition state in *Rm*Bgt17A are mutated in a conserved manner. Although the comparison between ΔG7048 and *Rm*Bgt17A provides molecular features for the transglucanase activity, the analysis of the ΔG7048 surface (in complex with a substrate modeled from *Rm*Bgt17A-LAM2/LAM5 complexes) suggested that G7048 may accommodate larger and branched substrates (Fig. 6c, d) also due to the absence of a disulfide bridge between C15 and C43 in the *Rm*Bgt17A structure. Since its catalytic cleft appears longer and larger in the region upstream of the -3-substrate binding site, it is likely that G7048 may act not only on linear substrates with a DP ≥ 5 but also on branched substrates. The latter conclusion is also corroborated by the capability of ΔG7048 to generate multimeric products from LAM5 (Additional File 2: Figure S6—*Right panel*). Although our analyses suggested the presence of 1,6-β-linkage(s) in the hybrid oligomers (Figs. 3, 4 and Additional File 2: Figure S8), additional experiments are required to finely characterize the type and localization of glycosidic linkage generated by the branching activity of G7048.

## Conclusions

The antagonizing activity between G9376 and G7048 suggests how opportunistic species belonging to *Penicillium* genus may feed on substrates with a composition similar to their own cell wall without incurring in self-deleterious autohydrolysis. Notably, enzymes belonging to the GH17 family are present only in fungi and plants [40]. These organisms are also the only 1,3-β-glucan-synthetizing organisms that can concomitantly produce 1,3-β-degrading enzymes, indicating that GH17/1,3-β-transglucanases might have evolved to preserve endogenous 1,3-β-glucan-based structures from the activity of their own 1,3-β-glucan degrading arsenal. Although glycoside hydrolase and transglycosylase activities can co-exist in the same enzyme [41, 42], transglucanases can use the degradation products from endo-glucanase activities, suggesting that the hydrolysis step might be a prerequisite for triggering their branching activity. The comprehension of mechanisms underlying 1,3-β-glucan metabolism is also important in view of the fact that, due to the incipient eutrophication processes, 1,3-β-glucan-rich biomass such as that from algae can be the next, valuable candidate for biofuel-related processes.

## Methods

### Design of the constructs expressing G9376 and ΔG7048 in *Pichia pastoris*

The amino acid sequences encoding the putative exo-1,3-β-glucanase G9376 and the putative glucan endo-1,3-β-glucosidase eglC G7048 from *Penicillium sumatraense* AQ67100 were reverse-translated in accordance with the codon usage of *Pichia pastoris* using the online tool OPTIMIZER (http://genomes.urv.es/OPTIMIZER/) [43]. Both signal peptides of G9376 and G7048 were identified using the SignalP-5.0 server (https://services.healthtech.dtu.dk/service.php?SignalP-5.0) and excluded from the manipulation. GPI-anchor prediction was performed using the online tool NetGPI-1.1 (https://services.healthtech.dtu.dk/service.php?NetGPI). Prediction of potential N-glycosylation sites was performed with the online tool GlycoEP by setting SVM threshold to 0.5 (https://webs.iiitd.edu.in/raghava/glycoep/submit.html). For the expression of truncated G7048, hereafter referred to as ΔG7048, the non-catalytic C-terminal domain (aa. 309–497; *see* Additional File 1: Data S1b) was excluded from the manipulation. The sequences encoding the restriction sites *Pst*I and *Xba*I were added at the 5^I^ and 3^I^ ends of each sequence and used to clone both genes in pPICZαB (Invitrogen, San Diego, CA). The sequences were synthesized by Genescript (https://www.genscript.com/) and fused in frame at the N-terminus to α-factor secretion signal from *Saccharomyces cerevisiae* for secretion in the culture medium, and at the C-terminus to the c-myc epitope (EQKLISEEDL) and the 6×His-tag. The correct frame between α-factor secretion signal and each synthetic gene was maintained by adding two extra bases downstream of the restriction site *PstI*.

### Heterologous expression of G9376 and ΔG7048 in *Pichia pastoris*

The constructs pPICZαB/g9376 and pPICZαB/Δg7048 were transformed in *Escherichia coli* DH5α (ThermoFisher, Waltham, USA) for plasmid amplification. Then, the constructs were linearized by *Sac*I and introduced in *P. pastoris* by electroporation according to [44]. Transformant colonies were selected on solid YPDS medium [1% (w/v) yeast extract, 2% (w/v) peptone, 2% (w/v) dextrose, 1 M Sorbitol] supplemented with zeocin (100 μg/mL), here used as antibiotic resistance marker. For proteins expression at small scale, several colonies of transformants were inoculated in 5 mL of YPD medium supplemented with zeocin (100 μg/mL) and incubated at 28 °C in a rotary shaker at 180 rpm for 72 h. The cultures were centrifuged and the cell pellets resuspended in 1.5 mL of Buffered Minimal Medium [BMM; 0.1 M K-phosphate (pH 6.0), 1.34% (w/v) YNB, 4 × 10^–5^% (w/v) biotin and 0.5% (v/v) methanol] to induce the expression of G9376 and ΔG7048, then grown for additional 48 h. To detect the expression of recombinant proteins, the G9376 and ΔG7048 filtrates were evaluated by SDS–PAGE and immuno-decoration analysis using a monoclonal anti-HIS antibody (AbHis, Bio-rad, Hercules, USA). For large scale purification of G9376 and ΔG7048, 500 mL of 48 h-grown BMM cultures were used. After cell removal by centrifugation (2000×*g*, 10 min), the culture filtrate was filtered by a sterile Polyether Sulfone (PES) filter (0.2 μm cut off diameter) and 10×-concentrated using a modular tangential flow system (Vivaflow^®^ 200, Sartorius Stedim, Gottinga, Germany) with a cutoff diameter of 30 kDa and 10 kDa for G9376 and ΔG7048, respectively. Purification was performed by the immobilized metal affinity chromatography (IMAC) using a HiTrap S-sepharose column (GE Healthcare, USA) pre-equilibrated with 50 mM Tris–HCl pH 7.4, 0.5 M NaCl and 5 mM imidazole. Elution was performed by a linear gradient of buffer constituted by 50 mM Tris–HCl pH 7.4, 0.5 M NaCl and 0.5 M imidazole. The eluted fractions were assayed by immuno-decoration analyses and those containing the highest amounts of recombinant proteins were pooled and concentrated. The proteins were analyzed by SDS–PAGE/Coomassie blue staining to assess their purity. Concentrations of G9376 and ΔG7048 were measured by UV-absorbance at 280 nm [ε_G9376_ = 135000/(M*cm), ε_ΔG7048_ = 66,015/(M*cm)] using NanoDrop One spectrophotometer (Thermo Scientific, USA). The purified enzymes were used for all further analyses.

### Circular dichroism spectroscopy

Circular dichroism (CD) experiments were performed using a Jasco J-815 instrument (Jasco Inc., Easton, MD, USA), equipped with a Peltier thermoregulation system for temperature control. Experiments for ΔG7048 (0.11 mg/ml) and G9376 (0.3 mg/ml) were carried out in 30 mM sodium acetate pH 5.5, 50 mM NaCl. Static spectra were collected at 20 °C in the Far-UV region (200–250 nm) using a quartz cell with 1 mm optical path length (Hellman, Plainview, NY, USA) at scanning speed of 200 nm/min with a data pitch of 0.5 nm. Each spectrum was the average of three acquisitions. Thermal denaturation experiments were performed by raising the temperature by 1 °C/min from 25 to 75 °C and monitoring the variation in CD signal at 225 nm (ΔG7048) and 217 nm (G9376) in 30 mM sodium acetate pH 5.5, 50 mM NaCl. Kaleidagraph (Synergy) software were used for graphing and data analysis.

### Enzymatic characterization of G9376

The enzymatic characterization of G9376 was performed using different chromogenic activity assays. Specific activity was evaluated by incubating G9376 (30 nM) in a buffer composed of 50 mM Na-Acetate pH 5 and 25 mM NaCl at 25 °C, and in the presence of the following substrates: 5 mM *p*-nitrophenyl-β-glucopyranoside (*p*NPGlc), 5 mM *p*-nitrophenyl-β-galactopyranoside (*p*NPGal), 5 mM *p*-nitrophenyl-β-laminaribioside (*p*NPLAM2), 0.5% (w/v) polygalacturonic acid (PGA), 1% (w/v) carboxymethyl-cellulose (CMC), 1% arabinoxylan (AX), 0.2% (w/v) 1,3-β-D-laminaripentaitol borohydride (LAM5ol), 0.2% (w/v) laminarin from *Laminaria digitata*, 0.1% (w/v) laminaribiose (LAM2), 0.1% (w/v) laminaritriose (LAM3), 0.1% (w/v) laminaritetraose (LAM4), 0.1% (w/v) laminaripentaose (LAM5) and 0.1% (w/v) laminarihexaose (LAM6). In parallel, the substrate specificity of ΔG7048 was evaluated by incubating the enzyme (320 nM) under the same experimental conditions but no significant activities were detected. *p*NPGlc, *p*NPGal and laminarin from *L. digitata* (N9634) were purchased from Sigma-Aldrich (Saint Louis, USA), whereas *p*NPLAM2, PGA, CMC, AX, LAM5ol and all 1,3-β-glucan oligomers (LAM2–6) were purchased from Megazyme (Dublin, Ireland). For the production of LAMol-oligomers, LAM3 and LAM4 were separately dissolved (10 mg/mL) in a freshly prepared solution of NaBH_4_ (130 mM) and incubated at 25 °C. After 2.5 h of incubation, the samples were diluted by adding 9 volumes of a filtered buffer composed of 50 mM Na-Acetate pH 5 and 25 mM NaCl, and used as LAMol standards (1 mg/ml) in HPLC analysis. pH- and temperature-dependent activities were determined by incubating G9376 (30 nM) in the presence of 0.2% (w/v) laminarin from *L. digitata*. Buffers at different pH values ranging from 4 to 9 were supplied with 25 mM NaCl and used for evaluating the pH optimum, whereas the temperature optimum was determined by incubating the enzymatic reactions (pH 5.0) at temperatures ranging from 20 °C to 60 °C. The enzymatic thermostability was tested at pH 5.0 after 1 h pre-incubation of G9376 (30 nM) at different temperatures ranging from 20 °C to 80 °C. Specific activity toward LAM5ol was assayed by incubating G9376 (30 nM) at pH 5.0 and 25 °C. Enzyme activity was expressed as Units (μmol of reducing sugar equivalents released per minute, or μmol of D-Glucose (Glc) released per minute, or μmol of *p*-nitrophenol released per minute) per mg of enzyme. Enzyme activities were calculated at three different timepoints from the linear phase of each reaction, converted into Units and then averaged. Relative activity (%) was calculated at each pH or *T* value as [Units at (pH or *T*)_x_/Units at (pH or *T*)_opt_] ×100% to obtain the pH- or *T*-dependent activity curves. The amount of reducing ends and Glc released upon hydrolysis were determined by *p*-hydroxybenzoic acid hydrazide assay (reducing sugar assay) according to [45] and by the commercial glucose oxidase–peroxidase assay kit (GO-POD assay) (Megazyme, Dublin, Ireland), respectively, using different amounts of Glc as calibration curve. The amount of *p*-nitrophenol released upon hydrolysis was determined using different amounts of *p*-nitrophenol as calibration curve.

### Analysis of degradation products from G9376 and ΔG7048 activities by HPLC

The analysis of degradation products from G9376 and ΔG7048 activities was performed by HPLC. Sugar standards were dissolved at 1 mg/mL (Glc, LAM2–6) and 2 mg/mL (laminarin) in a filtered buffer composed of 50 mM Na-Acetate pH 5 and 25 mM NaCl, and analyzed by HPLC. For the analysis of degradation products, dissolved LAM2-6 were separately incubated in the presence of G9376 (30 nM) for 1 h or, alternatively, in the presence of ΔG7048 (320 nM) for 24 h. For the analysis of degradation products, dissolved laminarin was incubated in the presence of G9376 (30 nM) for 1 h and 24 h. For the treatment with both enzymes, LAM5 was first incubated with ΔG7048 for 24 h (referred to as ΔG7048-pretreated LAM5) and then with G9376 for 1 h. All the reactions were performed at 25 °C unless otherwise indicated. In the reaction [ΔG7048-pretreated LAM5] (Table 2), the amount of hybrid oligosaccharides was calculated as the difference between the starting amount of LAM5 (10.23 µg) and the amount of degradation product LAM2. In the reaction [ΔG7048-pretreated LAM5 + G9376] (Table 2), the amount of degradation by-product X was calculated as the difference between the starting amount of LAM5 (10.23 µg) and the amount of degradation products LAM2 and Glc. Here, the amounts of different degradation products were converted also into nanomoles using the following molar mass values: 180 g/mol (Glc), 342 g/mol (LAM2), 504 g/mol (degradation by-product X, i.e., trimer) and 1315 g/mol (hybrid oligosaccharide, i.e., octamer).

HPLC analysis was carried out using a Shimadzu LC-2030 Plus Prominence-i (Japan) system equipped with a Shimadzu Differential Refractive Index Detector (RID-20A). Chromatographic separation was carried out using an Eurokat-Pb column 10 µm (300×4 mm, Knauer) with isocratic elution using distilled water (referred to as eluent A). The injection volume for all samples was 10 µL, whereas the flow rate and run time were 0.4 mL/min and 15 min, respectively. The eluent A was filtered through 0.2 μm pore size filter. The column was maintained at 75 °C throughout analysis, and the RID detector was set at 30 °C. All the data acquired were processed by Shimadzu LabSolutions control software.

The analysis of the degradation products obtained using LAM5ol as a substrate of G9376 was performed by HPAEC using an ICS3000 system (Dionex Thermo Fisher, https://www.thermofisher.com) equipped with a pulsed amperometric detector (PAD) using a gold electrode with waveform A, according to the manufacturer’s instructions. Dissolved LAM5ol was incubated in the presence of G9376 (30 nM) for 15, 45, 90 and 180 min, then the mixtures were heated at 90 °C for 10 min. The samples (10 µl) were injected on a CarboPac PA200 3 × 250 mm analytical column with a guard column (Thermo Fisher) kept at 35 °C. The flow rate was 0.4 ml/min and eluents A (0.05 M NaOH) and B (1 M Na-acetate in 0.05 M NaOH) were applied as follows after injection: a 5 min linear gradient to 2% B and then to 10% B in 8 min, followed by a 2 min linear gradient to 25% B and a 1 min gradient to 70% B. 100% B was kept for 5 min before returning to 100% A and equilibrating for 10 min.

### Analysis of enzymatic products from ΔG7048 activity by mass spectrometry.

For MALDI–MS analysis, the matrix solution was prepared by dissolving 2,5-dihydroxybenzoic acid (DHB) in a solution of 70% acetonitrile (MeCN) with 0.1% trifluoroacetic acid (TFA) to a final concentration of 20 mg/mL. The samples (1 mg/ml in water) were pre-treated for 10 min with BioRex MSZ501 cation exchange resin beads (BIO-RAD) and then prepared as dried-droplets by spotting 1 μL of matrix solution first on the stainless steel MALDI target and adding 1 μL of sample solution immediately afterward. Samples were allowed to dry under ambient conditions for 30 min. As additional control, laminarioctaose (LAM8) was dissolved at 1 mg/mL in a filtered buffer composed of 50 mM Na-Acetate pH 5 and 25 mM NaCl, pre-treated for 10 min with BioRex MSZ501 cation exchange resin beads and then analyzed. LAM8 was purchased from Megazyme (Dublin, Ireland). MALDI–TOF–MS measurements were performed on an UltrafleXtreme TOF/TOF mass spectrometer equipped with a reflector and controlled by the FlexControl 2.2 software package (Bruker Daltonics).

### Crystallization and data collection of ΔG7048

ΔG7048 crystallization conditions were initially screened automatically using the Crystal Phoenix robot (Art Robbins instruments). Small single crystals were observed in the condition n. 28 of the Crystal screen 2 (Hampton Research) and were further optimized by the hanging drop vapor diffusion method. Best diffracting crystals grew in 2–5 days and were obtained by mixing 1 μL of protein solution (4 mg/mL) with 1.5 μL of the reservoir solution containing 0.1 M Tris–HCl pH 7.5, and 1.4 M Sodium Citrate dihydrate tribasic and equilibrated versus 500 μL of reservoir solution at 20 °C. Crystals were cryoprotected with 20% glycerol, flash-frozen and exposed to X-ray at the XRD2 Beamline of ELETTRA Synchrotron (Trieste, Italy). Diffraction data, collected at a wavelength of 1.000 Å with an oscillation of 0.5°, were processed and scaled with XDS [46] and AIMLESS [47].

### ΔG7048 structure solution and refinement

The structure was solved at resolution 1.9 Å by molecular replacement with Phaser [48] as implemented in Phenix [49] using the structure of Bgt17A from *R. miehei* (pdb code: 4wtp, [26]) as the search model. The initial model was subjected to several cycles of refinement with Phenix_refine [50] and manual visual inspection and rebuilding using COOT [51]. The final model was refined to a *R*_work_ and a *R*_free_ of 0.16% and 0.20%, respectively (Table 3) and contains 290 residues; residues 35 and 36 could not be fitted in the electron density, while the *N*-glycosylation chain composed of two NAG, one BMA, and four MAN was added to Asn166. Statistics for data collection and refinement are reported in Table 3 Coordinates and structure factors have been deposited in the Protein data bank with accession code 8akp. Figures were prepared with Chimera [52].

## Supplementary Information

**Additional file 1: Data S1.** Amino acid sequences of G9376 and G7048 from *P. sumatraense *AQ67100. **Data S2.** Amino acid sequences of the two putative β-1,3-modifying enzymes from *P. sumatraense *AQ67100 as expressed in *P. pastoris*.

**Additional file 2: Figure S1.** Purification of recombinant G9376 and ΔG7048 from *P. pastoris. ***Figure S2.** Far-UV CD analysis of G9376 and ΔG7048. **Figure S3.** Analysis of degradation products obtained from two different 1,3-β-glucan pentamers upon incubation with exo-1,3-β-glucanase G9376. **Figure S4.** HPAEC–PAD analysis of degradation products obtained from LAM5ol upon incubation with exo-1,3-β-glucanase G9376. **Figure S5.** Production kinetic of the hybrid oligosaccharide generated by 1,3-β-transglucanase ΔG7048 using LAM5 as substrate. **Figure S6.** Mass spectrometry analysis of the hybrid oligosaccharides as generated by the activity of 1,3-β-transglucanase ΔG7048 on LAM5. **Figure S7.** Structural superposition of ΔG7048 with *Rm*Bgt17A. **Figure S8.** Amino acid alignment between the catalytic domains of 1,3-β-transglucanase ΔG7048 from *P. sumatraense* AQ67100 and Bgt2p from *A. fumigatus*.

## Data Availability

All relevant data are included in the article and/or its Additional files. The data sets used and/or analyzed during the current study are available from the corresponding authors on reasonable request. Coordinates and structure factors of the crystallographic structures have been deposited in the Protein Data Bank (PDB) with accession code 8akp.

## Abbreviations

AX: Arabinoxylan
CAZy: Carbohydrate-active enzyme
CMC: Carboxymethyl-cellulose
CWDE: Cell wall-degrading enzyme
DP: Degree of polymerization
GHn: Glycoside hydrolase family n
Glc: D-Glucose
LAM2: Laminaribiose
LAM3: Laminaritriose
LAM4: Laminaritetraose
LAM5: Laminaripentaose
LAM2ol: 1,3-β-D-laminaribiitol borohydride
LAM3ol: 1,3-β-D-laminaritriitol borohydride
LAM4ol: 1,3-β-D-laminaritetraitol borohydride
LAM5ol: 1,3-β-D-laminaripentaitol borohydride
LAM6: Laminarihexaose
LAM8: Laminarioctaose
PGA: Polygalacturonic acid
*p*NPGlc: *p*-Nitrophenyl-β-glucopyranoside
*p*NPGal: *p*-Nitrophenyl-β-galactopyranoside
*p*NPLAM2: *p*-Nitrophenyl-β-laminaribioside
TIM-barrel: Triose-phosphate isomerase-barrel
