# Supplementary material for: Characterization of two 1,3-β-glucan-modifying enzymes from Penicillium sumatraense reveals new insights into 1,3-β-glucan metabolism of fungal saprotrophs
Source: Biotechnol Biofuels Bioprod. 2022 Dec 12;15:138. doi: 10.1186/s13068-022-02233-8 (PMC9745967; doi:10.1186/s13068-022-02233-8)
Supplement: Supplementary file 1 — Additional file 1: Data S1. Amino acid sequences of G9376 and G7048 from P. sumatraense AQ67100. Data S2. Amino acid sequences of the two putative β-1,3-modifying enzymes from P. sumatraense AQ67100 as expressed in P. pastoris. [file 13068_2022_2233_MOESM1_ESM.pdf]

Additional file 1: Data S1. Amino acid sequences of G9376 and G7048 from *P. sumatraense* AQ67100. Amino acid sequence of the predicted a) “1,3-β-exoglucanase” G9376 and b) “Glucan endo-1,3-β-glucosidase eglC” G7048 from *P. sumatraense* AQ67100 (Giovannoni et al., 2021). [yellow amino acids, predicted signal peptide sequence; green amino acids, non-catalytic C-terminal sequence of G7048; red amino acid: predicted ω site for the binding of G7048 to the GPI-tail].

a)

MHFASAITLVSLVSSVHTQLLEIPAVDELVSSALQPLEAWTDYQGPTGIASSALSKSTHAIV
ANVAVEAADASYWLADISHQGKAAFNPNPSSYKVFRNVKDYGAKGDGVTDDTAAINSAI
SDGGRYGPSSRQSSTTTPAIVYFPAGTYLISTPIIDYYFTQLIGNPNSMPVIKATAGFSGLGLI
DGDQYQSDGNQGWTSTNVFFRQIRNLKLDLTNIPASSAATGIHWPTGQATSIQNVDIVMSS
ASGTQHQGIFIENGSGGFLADITITGGLYGANVGNQQFTMRNLVITDAVTAISQIWDWGWT
YQGLTVTNCSTALSVDNGGAGNQLVGSVIVLDSTIQDCSTFVTSAWQASTFSNGSLILENIS
LENVPVAVKGPSGTVLVGGTMTISAWGQGHKYTPNGPTNFQGTFTAPTRPSSLLASGSSRY
YTKSKPQYETLSQSSFVSTRSAGATGDGSTDDTSAIQSALNSAASSGKIVFFDQGTYKVTDT
IYVPPGSRITGEAYPVIMASGSAFSSISKPVPVVQVGKSGESGSVEWSDMIVSTQGSTPGAVL
IEWNLAANSGSGMWDVHTRIGGFSGSQQQVAQCPTSAAVSAACEVAYMSMHITDSASGV
YLDNVWLWTADHDLDSADNTRISVYSGRGLLIEGQTIWLYGTGVEHHSLYQYQFSGASSV
VAGFIQTETPYYQPNPDAANGPYPSNPDLKDPDYSSCLSGNCDSLGLRVLDSSDIVIYGAGL
YSFFNNYSTDCSTFPVPENCQSEIFSIEGDTSNLVVYALSTVGTTNMIVKDGTSLAVVSDNL
ATYAATIAYFTL

b)

MQMIQLLALALSVATADAVSKGFNYGATKADGSSKYQADFKKDFAAAKALVEGGSGFTS
ARLYTMIQGGTTNTPIEAIPAAIEEKTELLLGLWASGGNMDNEIAALKSAISQYGDDFANLV
VGISVGSEDMYRNSVTGSKSNAGPGVEPEELVSYIQQVRSTIAGTGLSDASIGHVDTWDSW
TNSSNSDVVNHLDWLGFDGYPYYQLTMENGIENAKKLFDESVEKTKSVANGKEVWITET
GWPVTGPQEGDATASPANAKTYWDEVGCPLFGNTNTWWYMLEDEGASPSFGVVKSDLK
TPQFDLSCSNSGSSTSSSTAAGSSSTGSISNKDSTGSGSNSGSGSGSSSDSNSGSSSSSAGSSSS
SGSSSGSSSASASGSGSTSGSASGSSSSPAVSSKPTFSAGRNGTHSSSFQSASRTSTPLTSATG
SGSGSGSGSSSSGSGSSSGAGSSSGSSDSGSASGSAASPSSTFNAAIRSSGAGSTFGAIVAAA
VLAIAV

SAdditional file 1: Data S2. Amino acid sequences of the two putative β-1,3-modifying enzymes from *P. sumatraense* AQ67100 as expressed in *P. pastoris*. Amino acid sequence of a) G9376 and b) truncated ΔG7048 as expressed in *P. pastoris*. Each sequence was fused at the N-terminus to α-factor secretion signal (in yellow) and at the C-terminus to *c*-myc epitope (in green) and 6xhis-tag (in turquoise). Potential N-glycosylation sites of mature proteins are highlighted in purple.

a)

MRFPSIFTAVLFAASSALAAPVNTTTEDETAQIPAEAVIGYSDLEGDFDVAVLPFSNSTNNG
LLFINTTIASIAAKEEGVSLEKREAEAAAQLLEIPAVDELVSSALQPLEAWTDYQGPTGIASS
ALSKSTHAIVANVAVEAADASYWLADISHQGKAAFNPNPSSYKVFRNVKDYGAKGDGVT
DDTAAINSAISDGGRYGPSSRQSSTTTPAIVYFPAGTYLISTPIIDYYFTQLIGNPNSMPVIKAT
AGFSGLGLIDGDQYQSDGNQGWTSTNVFFRQIRNLKLDLTNIPASSAATGIHWPTGQATSIQ
NVDIVMSSASGTQHQGIFIENGSGGFLADITITGGLYGANVGNQQFTMRNLVITDAVTAISQ
IWDWGWTYQGLTVTNCSTALSVDNGGAGNQLVGSVIVLDSTIQDCSTFVTSAWQASTFSN
GSLILENISLENVPVAVKGPSGTVLVGGTMTISAWGQGHKYTPNGPTNFQGTFTAPTRPSSL
LASGSSRYYTKSKPQYETLSQSSFVSTRSAGATGDGSTDDTSAIQSALNSAASSGKIVFFDQ
GTYKVTDTIYVPPGSRITGEAYPVIMASGSAFSSISKPVPVVQVGKSGESGSVEWSDMIVST
QGSTPGAVLIEWNLAANSGSGMWDVHTRIGGFSGSQQQVAQCPTSAAVSAACEVAYMSM
HITDSASGVYLDNVWLWTADHDLDSADNTRISVYSGRGLLIEGQTIWLYGTGVEHHSLYQ
YQFSGASSVVAGFIQTETPYYQPNPDAANGPYPSNPDLKDPDYSSCLSGNCDSLGLRVLDS
SDIVIYGAGLYSFFNNYSTDCSTFPVPENCQSEIFSIEGDTSNLVVYALSTVGTTNMIVKDGT
SLAVVSDNLATYAATIAYFTLALEQKLISEEDLNSAVDHHHHHH

b)

MRFPSIFTAVLFAASSALAAPVNTTTEDETAQIPAEAVIGYSDLEGDFDVAVLPFSNSTNNG
LLFINTTIASIAAKEEGVSLEKREAEAAAVSKGFNYGATKADGSSKYQADFKKDFAAAKAL
VEGGSGFTSARLYTMIQGGTTNTPIEAIPAAIEEKTELLLGLWASGGNMDNEIAALKSAISQ
YGDDFANLVVGISVGSEDMYRNSVTGSKSNAGPGVEPEELVSYIQQVRSTIAGTGLSDASI
GHVDTWDSWTNSSNSDVVNHLDWLGFDGYPYYQLTMENGIENAKKLFDESVEKTKSVA
NGKEVWITETGWPVTGPQEGDATASPANAKTYWDEVGCPLFGNTNTWWYMLEDEGASP
SFGVVKSDLKTPQFDLSCSAEQKLISEEDLNSAVDHHHHHH
