# Supplementary material for: Characterization of two 1,3-β-glucan-modifying enzymes from Penicillium sumatraense reveals new insights into 1,3-β-glucan metabolism of fungal saprotrophs
Source: Biotechnol Biofuels Bioprod. 2022 Dec 12;15:138. doi: 10.1186/s13068-022-02233-8 (PMC9745967; doi:10.1186/s13068-022-02233-8)
Supplement: Supplementary file 2 — Additional file 2: Figure S1. Purification of recombinant G9376 and ΔG7048 from P. pastoris. Figure S2. Far-UV CD analysis of G9376 and ΔG7048. Figure S3. Analysis of degradation products obtained from two different 1,3-β-glucan pentamers upon incubation with exo-1,3-β-glucanase G9376. Figure S4. HPAEC–PAD analysis of degradation products obtained from LAM5ol upon incubation with exo-1,3-β-glucanase G9376. Figure S5. Production kinetic of the hybrid oligosaccharide generated by 1,3-β-transglucanase ΔG7048 using LAM5 as substrate. Figure S6. Mass spectrometry analysis of the hybrid oligosaccharides as generated by the activity of 1,3-β-transglucanase ΔG7048 on LAM5. Figure S7. Structural superposition of ΔG7048 with RmBgt17A. Figure S8. Amino acid alignment between the catalytic domains of 1,3-β-transglucanase ΔG7048 from P. sumatraense AQ67100 and Bgt2p from A. fumigatus. [file 13068_2022_2233_MOESM2_ESM.pdf]

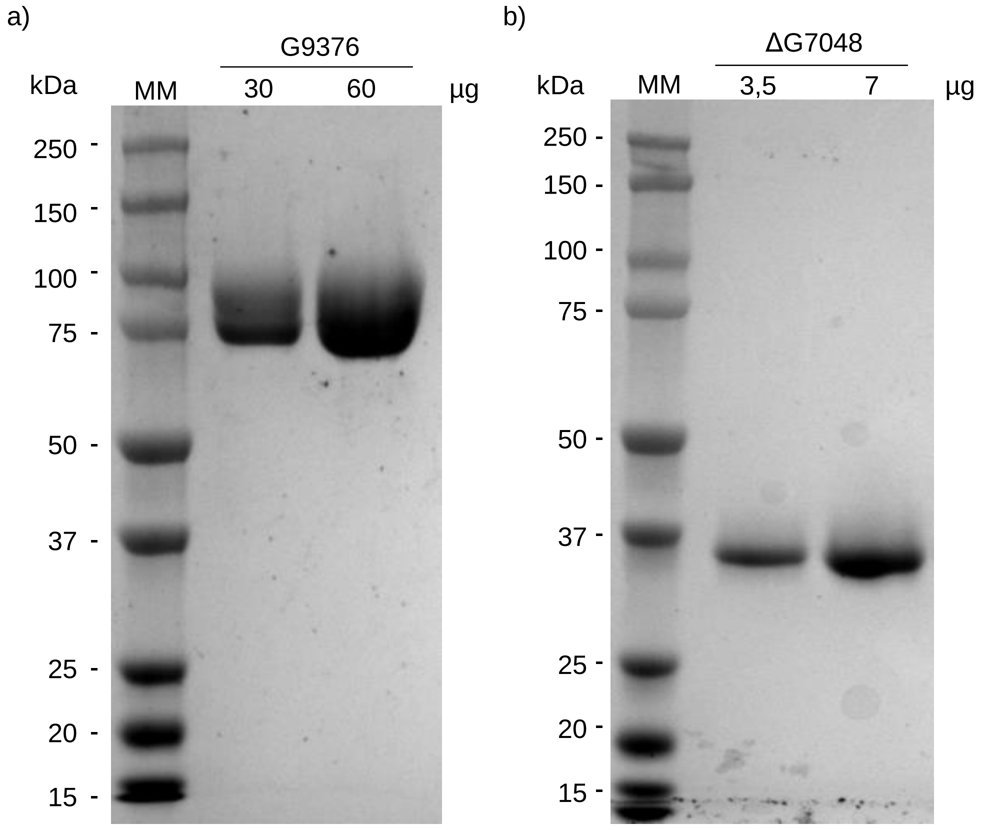

Supplementary Figure S1. Purification of recombinant G9376 and ΔG7048 from *P. pastoris*. SDS-page/Comassie blu staining analysis of a) G9376 and b) ΔG7048 as eluted from the IMAC chromatography. Different amounts (µg) were evaluated for each protein. Molecular weight marker (MM) is also reported.

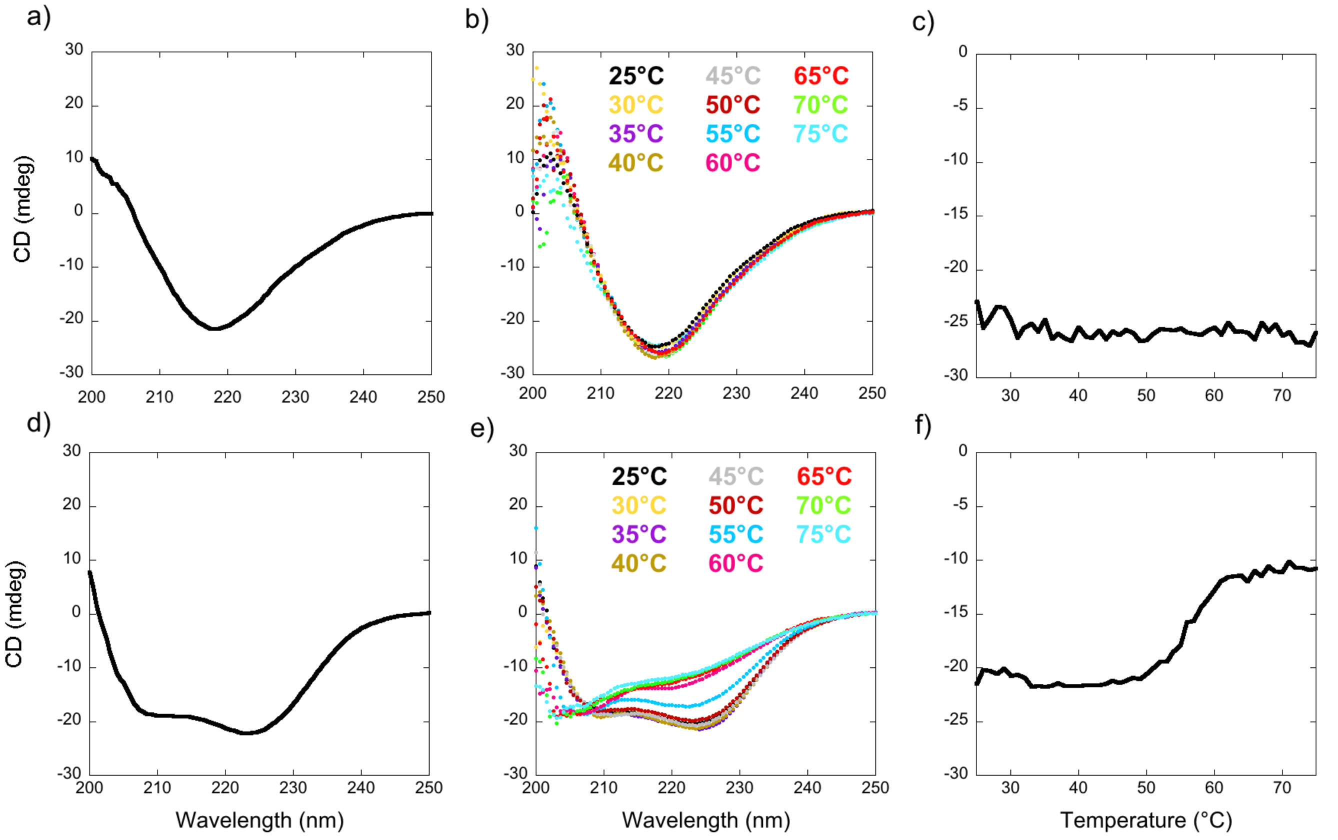

Supplementary Figure S2. Far-UV CD analysis of G9376 and ΔG7048. a-b) CD spectra of G9376 collected at a) 20°C and b) different temperatures ranging from 25°C to 75°C as represented by different colors (black, 25°C; yellow, 30°C; purple, 35°C; brown, 40°C; gray, 45°C; dark red, 50°C; light blue, 55°C; violet, 60°C; red, 65°C; light green, 70°C; cyan, 75C°). c) Thermal denaturation profile of G9376 obtained by monitoring the variation in CD signal at 217 nm. d-e) CD spectra of ΔG7048 collected at d) 20°C and e) different temperatures ranging from 25°C to 75°C as represented by the same colors used in (b). f) Thermal denaturation profile of ΔG7048 obtained by monitoring the variation in CD signal at 225 nm. Experiments were carried out in 30 mM sodium acetate pH 5.5 and 50 mM NaCl.

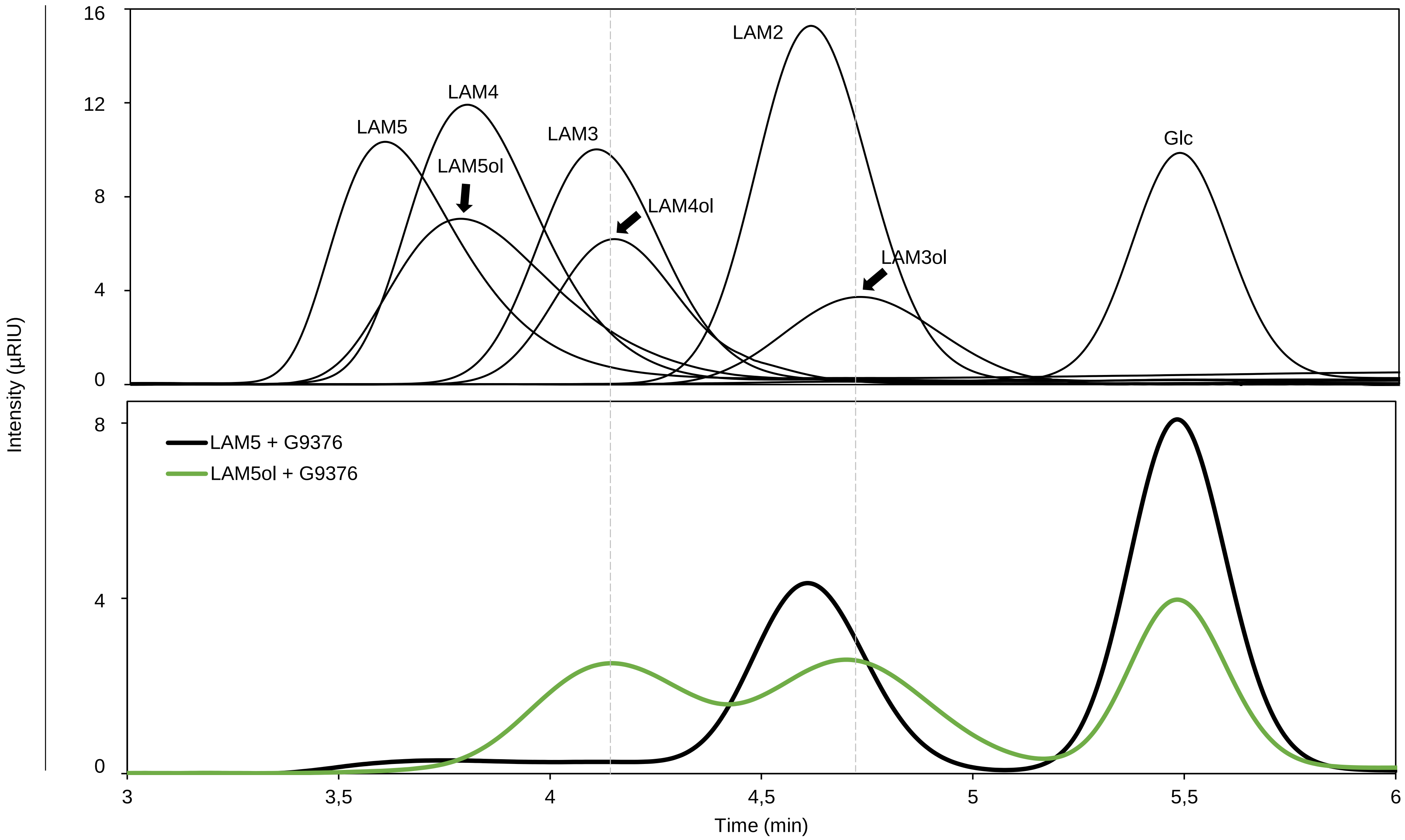

Supplementary Figure S3. Analysis of degradation products obtained from two different 1,3-β-glucan pentamers upon incubation with exo-1,3-β-glucanase G9376. Chromatographic analysis of (*upper panel*) different 1,3-β-glucan oligomers and (*lower panel*) of degradation products as obtained from 1,3-β-glucan pentamer (LAM5, black line) and 1,3-β-D-laminaripentaitol borohydride (LAM5ol, green line) upon 1.5-h incubation with exo-1,3-β-glucanase G9376. In the upper panel, glucose is also analysed. [Glc, D-Glucose; LAM2, laminaribiose; LAM3, laminaritriose; LAM4, laminaritetraose; LAM5, laminaripentaose; LAM3ol, 1,3-β-D-laminaritriitol borohydride; LAM4ol, 1,3-β-D-laminaritetraitol borohydride; LAM5ol, 1,3-β-D-laminaripentaitol borohydride].

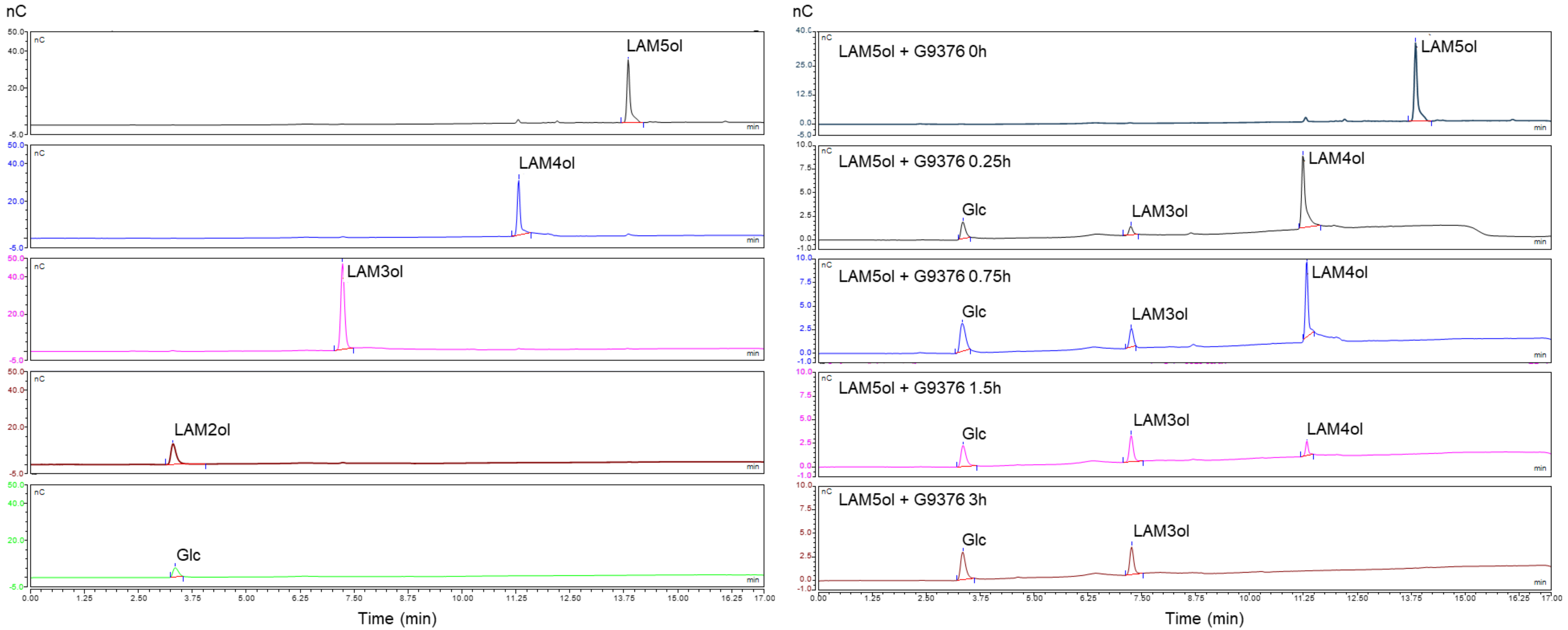

Supplementary Figure S4. HPAEC-PAD analysis of degradation products obtained from LAM5ol upon incubation with exo-1,3-β-glucanase G9376. Chromatographic analysis of (*left panels*) different 1,3-β-glucan-oligomers with borohydride-reduced C1-ends (LAMol oligomers) and (*right panels*) of degradation products as obtained from LAM5ol with exo-1,3-β-glucanase G9376 at different incubation times (0, 0.25, 0.75, 1.5 and 3h). [Glc, D-Glucose; LAM2ol, 1,3-β-D-laminaribiitol borohydride; LAM3ol, 1,3-β-D-laminaritriitol borohydride; LAM4ol, 1,3-β-D-laminaritetraitol borohydride; LAM5ol, 1,3-β-D-laminaripentaitol borohydride].

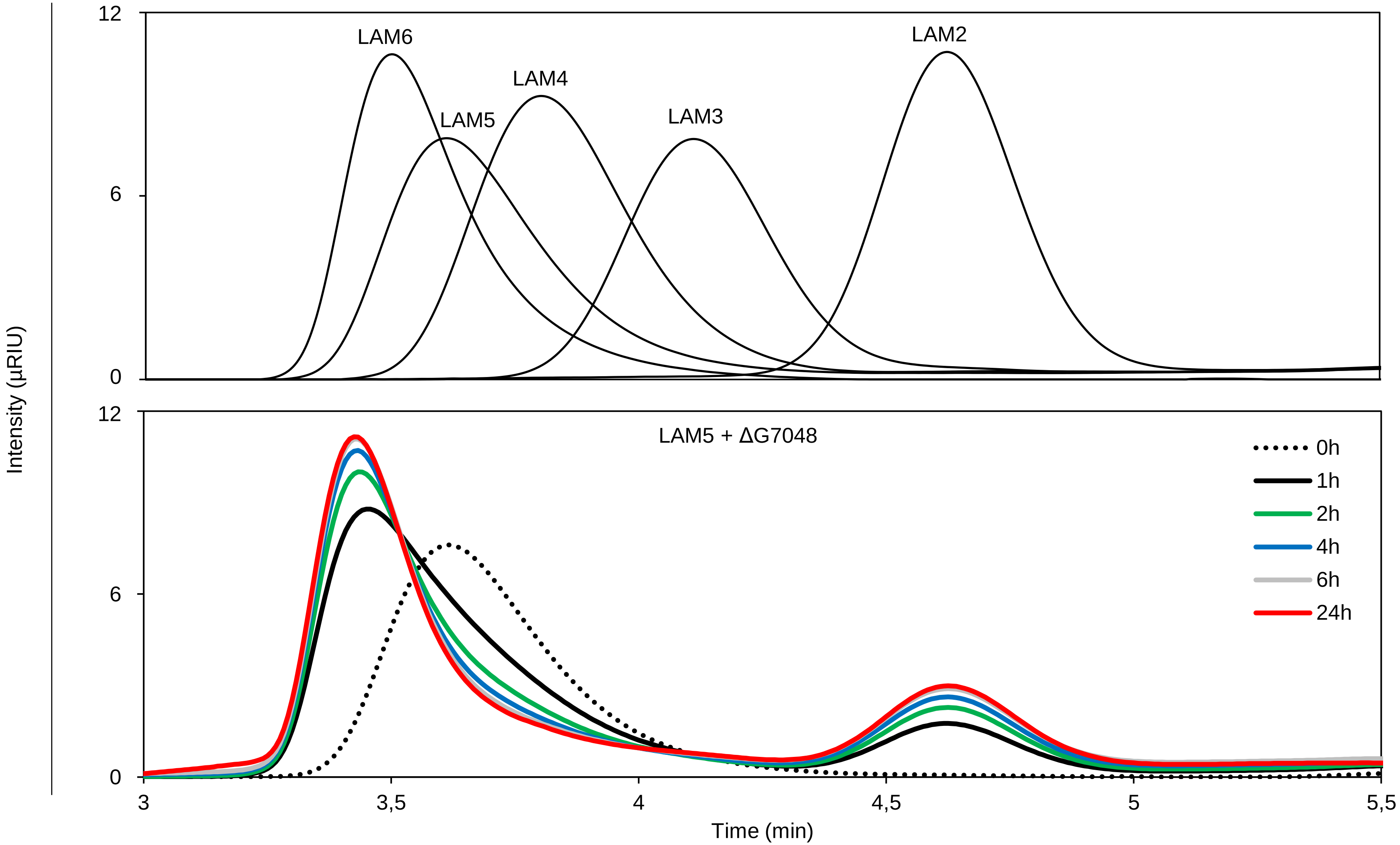

Supplementary Figure S5. Production kinetic of the hybrid oligosaccharide generated by 1,3-β-transglucanase ΔG7048 using LAM5 as substrate. Chromatographic analysis of (*upper panel*) different 1,3-β-glucan oligomers (LAM2-6) and of (*lower panel*) enzymatic products obtained from LAM5 upon different incubation times (0h, dotted line; 1h, black line; 2h, green line; 4h, blue line; 6h, grey line; 24h, red line) with 1,3-β-transglucanase ΔG7048. [LAM2, laminaribiose; LAM3, laminaritriose; LAM4, laminaritetraose; LAM5, laminaripentaose; LAM6, laminarihexaose].

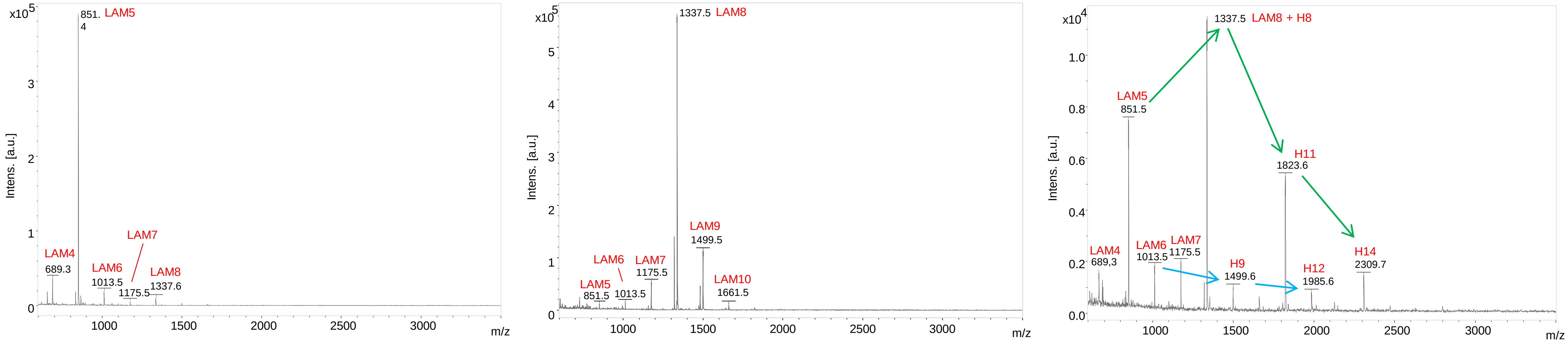

Supplementary Figure S6. Mass spectrometry analysis of the hybrid oligosaccharides generated by the activity of 1,3-β-transglucanase ΔG7048 on LAM5. MALDI-TOF mass spectrum of the commercial *left panel*) LAM5 (m/z = 851,4, [M + Na]$^+$) and *middle panel*) LAM8 (m/z = 1337,5, [M + Na]$^+$). *Right panel*) Products of the 24-hour incubation of LAM5 with the 1,3-β-transglucanase ΔG7048. Hybrid oligosaccharides are indicated as HX where X is the calculated degree of polymerization (DP). Green and turquoise arrows indicate two different +DP3-series. [HX, hybrid oligosaccharide with DPX; LAM4, laminaritetraose; LAM5, laminaripentaose; LAM6, laminarihexaose; LAM7, laminariheptaose; LAM8, laminarioctaose; LAM9, laminarinonaose; LAM10, laminarin-decamer].

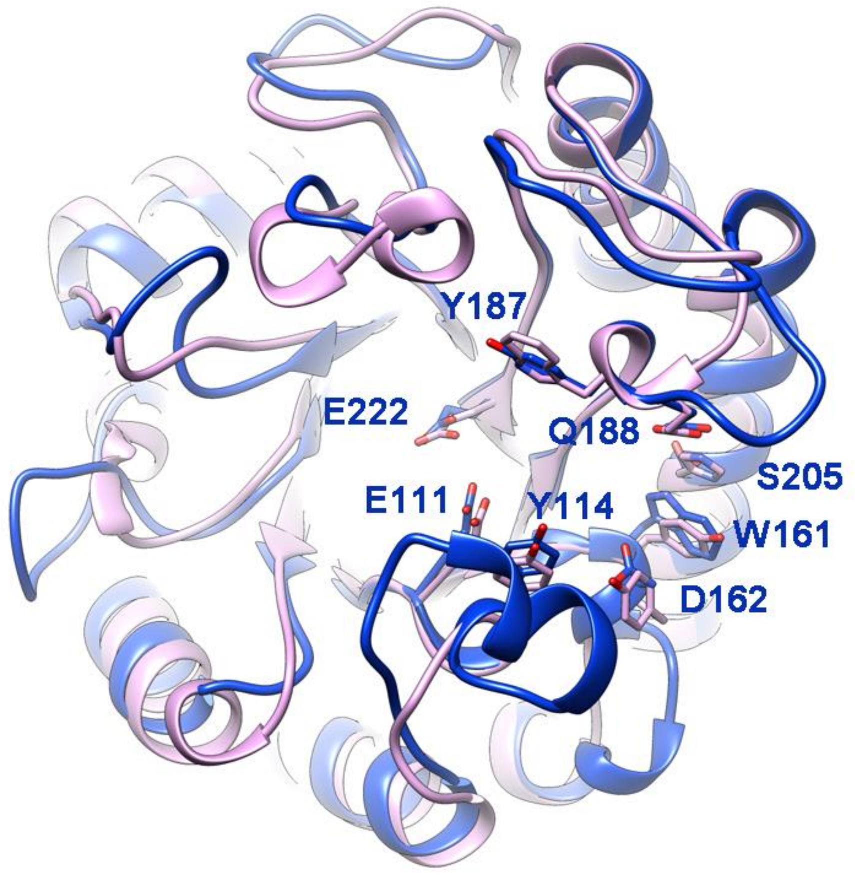

Supplementary Figure S7. Structural superposition of ΔG7048 with *Rm*Bgt17A. Structural superposition of ΔG7048 (blue) and *Rm*Bgt17A (PDB: 4wtp, pink) with relevant residues highlighted in stick representation: the catalityc residues (E111 and E222), residues crucial for transglycosylation activity (Y114, Y187 and Q188) and residues that create steric hindrance near the +2 glucosyl residue of the substrate, blocking the catalytic cleft of the enzyme (W161, D162, Q188 and S205). Numbering refers to the ΔG7048 structure.

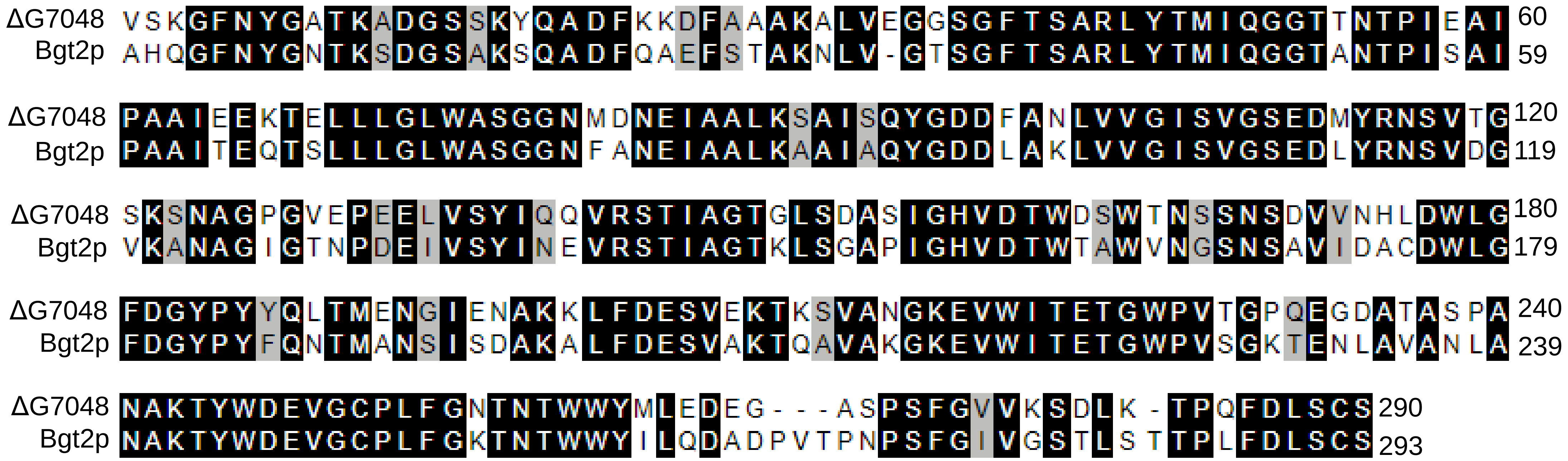

Supplementary Figure S8. Amino acid alignment between the catalytic domains of 1,3-β-transglucanase ΔG7048 from *P. sumatraense* AQ67100 and Bgt2p from *A. fumigatus*. Identical amino acids are in black background, whereas amino acid in grey background refers to amino acids with similar chemical properties. Numbering is from the first amino acid of each mature protein. The amino acid identity between the two proteins is 68.37%. The alignment was performed by excluding the non-catalytic C-terminal region of each protein, i.e., last 189 and 135 amino acids in ΔG7048 and Bgt2p, respectively. [ΔG7048, 1,3-β-transglucanase ΔG7048 from *P. sumatraense* AQ67100; Bgt2p, 1,3-β-glucanosyltransferase 2 from *Aspergillus fumigatus*].
